# Potent Allosteric Dengue Virus NS5 Polymerase Inhibitors: Mechanism of Action and Resistance Profiling

**DOI:** 10.1371/journal.ppat.1005737

**Published:** 2016-08-08

**Authors:** Siew Pheng Lim, Christian Guy Noble, Cheah Chen Seh, Tingjin Sherryl Soh, Abbas El Sahili, Grace Kar Yarn Chan, Julien Lescar, Rishi Arora, Timothy Benson, Shahul Nilar, Ujjini Manjunatha, Kah Fei Wan, Hongping Dong, Xuping Xie, Pei-Yong Shi, Fumiaki Yokokawa

**Affiliations:** 1 Novartis Institute for Tropical Diseases, Singapore; 2 School of Biological Sciences, Nanyang Technological University, Singapore; 3 UPMC UMRS CR7—CNRS ERL 8255-INSERM U1135 Centre d’Immunologie et des Maladies Infectieuses, Centre Hospitalier Universitaire Pitié-Salpêtrière, Faculté de Médecine Pierre et Marie Curie, Paris, France; 4 Novartis Institute for Biomedical Research, Cambridge, Massachusetts, United States of America; Washington University, UNITED STATES

## Abstract

Flaviviruses comprise major emerging pathogens such as dengue virus (DENV) or Zika virus (ZIKV). The flavivirus RNA genome is replicated by the RNA-dependent-RNA polymerase (RdRp) domain of non-structural protein 5 (NS5). This essential enzymatic activity renders the RdRp attractive for antiviral therapy. NS5 synthesizes viral RNA via a “*de novo”* initiation mechanism. Crystal structures of the flavivirus RdRp revealed a “closed” conformation reminiscent of a pre-initiation state, with a well ordered priming loop that extrudes from the thumb subdomain into the dsRNA exit tunnel, close to the “GDD” active site. To-date, no allosteric pockets have been identified for the RdRp, and compound screening campaigns did not yield suitable drug candidates. Using fragment-based screening via X-ray crystallography, we found a fragment that bound to a pocket of the apo-DENV RdRp close to its active site (termed “N pocket”). Structure-guided improvements yielded DENV pan-serotype inhibitors of the RdRp *de novo* initiation activity with nano-molar potency that also impeded elongation activity at micro-molar concentrations. Inhibitors exhibited mixed inhibition kinetics with respect to competition with the RNA or GTP substrate. The best compounds have EC_50_ values of 1–2 μM against all four DENV serotypes in cell culture assays. Genome-sequencing of compound-resistant DENV replicons, identified amino acid changes that mapped to the N pocket. Since inhibitors bind at the thumb/palm interface of the RdRp, this class of compounds is proposed to hinder RdRp conformational changes during its transition from initiation to elongation. This is the first report of a class of pan-serotype and cell-active DENV RdRp inhibitors. Given the evolutionary conservation of residues lining the N pocket, these molecules offer insights to treat other serious conditions caused by flaviviruses.

## Author Summary

Dengue virus (DENV) is the world’s most prevalent mosquito-borne viral disease and nearly 40% of the world’s population is at risk of infection. Currently, no specific drugs are available to treat dengue or other flaviviral diseases. DENV NS5 is a large protein of 900 amino acids composed of two domains with key enzymatic activities for viral RNA replication in the host cell and constitutes a prime target for the design of anti-viral inhibitors. We performed a fragment-based screening by X-ray crystallography targeting the DENV NS5 polymerase and identified an allosteric binding pocket at the base of the thumb subdomain close to the enzyme active site. Potent inhibitors active in both DENV polymerase biochemical and cell-based assays were developed through structure-guided design. Resistant virus replicons grown in the presence of the inhibitor, harbored amino acid changes that mapped to the compound binding site. The proposed mode of action for this class of inhibitors is by impeding RdRp protein conformational changes during the transition from initiation to elongation phase of enzyme activity.

## Introduction

Several flaviviruses, such as DENV, Japanese Encephalitis virus (JEV), West Nile virus (WNV), Yellow Fever virus (YFV) or Tick-borne encephalitis virus (TBEV) are major human pathogens, whilst Zika (ZIKV) is an emerging flavivirus of global significance causing severe neurological conditions in infected adults and newborn babies, most likely by mother-to-child transmission [1]. The mosquito-borne DENV causes widespread epidemics in over 100 countries, with ∼390 million infections each year [2]. Infection by any of the four DENV serotypes can lead to several outcomes, ranging from asymptomatic infection, dengue fever, to dengue hemorrhagic fever and dengue shock syndrome. After several decades of efforts, the first vaccine was recently licensed for use, but confers only partial cross protection for the four DENV serotypes [3, 4]. No antivirals have been approved to treat dengue or other flaviviral diseases [5].

Flavivirus RNA replication occurs in host cells on endoplasmic reticulum-derived membranes within a multi-protein replication complex (RC) consisting of viral NS proteins and host cofactors [6–8]. Comprising 900 amino acid residues, NS5 is the largest and most conserved protein component of the flavivirus RC. Its N-terminal domain (residues 1–265 in DENV3) is an S-adenosyl-L-methionine (SAM)-dependent methyltransferase (MTase) that methylates the viral RNA genome cap [9–15]. A guanylyltransferase activity was also proposed for the N-terminal domain of NS5 [16, 17]. Its C-terminal RdRp domain (residues 267–900) synthesizes the viral genomic RNA [18–22]. A potentially flexible linker region that connects the two catalytic domains of NS5 regulates RdRp activities and virus replication by modulating MTase-RdRp interactions [23–25]. In addition to its enzymatic functions, NS5 inhibits host interferon-mediated signaling by promoting degradation of STAT2 [26]. In DENV, NS5 localizes to the nucleus of infected cells in a serotype-dependent manner that modulates host processes [27].

Following DENV infection, the RdRp synthesizes viral RNA in the absence of a primer strand, via a *de novo* initiation mechanism, in which the (+) strand viral RNA template is transcribed into a complementary RNA strand of (-) polarity [18, 19]. This duplex in turn serves as a template for synthesis of additional RNA strands of (+) polarity that either act as mRNA for protein translation or are packaged into virions. DENV RdRp possesses a right hand-like architecture conserved across different polymerase families [21, 22, 25], with three subdomains termed ‘‘fingers”, ‘‘palm” and ‘‘thumb”. Within these subdomains, seven conserved amino-acid sequence motifs play key roles for binding RNA, NTPs and metal-ions and for catalysis [28, 29]. Structures of the apo-DENV RdRp were found to adopt a “closed” pre-initiation state conformation, with a well-ordered priming loop projecting into a narrow RNA binding tunnel. Disordered peptide segments were observed in motifs F, G and at the C-terminal end [21, 22, 25].

The importance of NS5 for viral replication makes it an ideal target for developing inhibitors to treat diseases caused by flaviviruses [30–32]. Although several high-throughput screening campaigns have been performed, only a few DENV RdRp non-nucleoside inhibitors have been described [33–36]. From these latter efforts, we previously identified two compounds that bind to the RNA tunnel but did not succeed in improving their lead-like properties [34, 35]. Here, using fragment-based screening via X-ray crystallography targeting the apo-DENV RdRp, we identified a fragment that bound to a pocket located in the thumb subdomain, close to the enzyme active site, which we term as the “N pocket” [37, 38]. Using a structure-guided approach that combines biochemical, biophysical and cell-based assays, we designed potent inhibitors that bound to this allosteric site, and inhibited DENV1-4 viral replication across various cell-based assays. Resistant DENV replicons with amino acid changes in the “N” pocket were raised with two compounds, confirming that the NS5 polymerase was the specific target for this class of inhibitors in DENV infected cells. To our knowledge, this is the first report of a Flavivirus RdRp allosteric pocket and the successful use of structure-guided approach for designing potent inhibitors targeting NS5. This work has major implications for the design of much-needed flavivirus anti-viral inhibitors.

## Results

### Structure-guided design of a novel chemical scaffold that binds to the DENV RdRp N pocket

**Fig 1:**
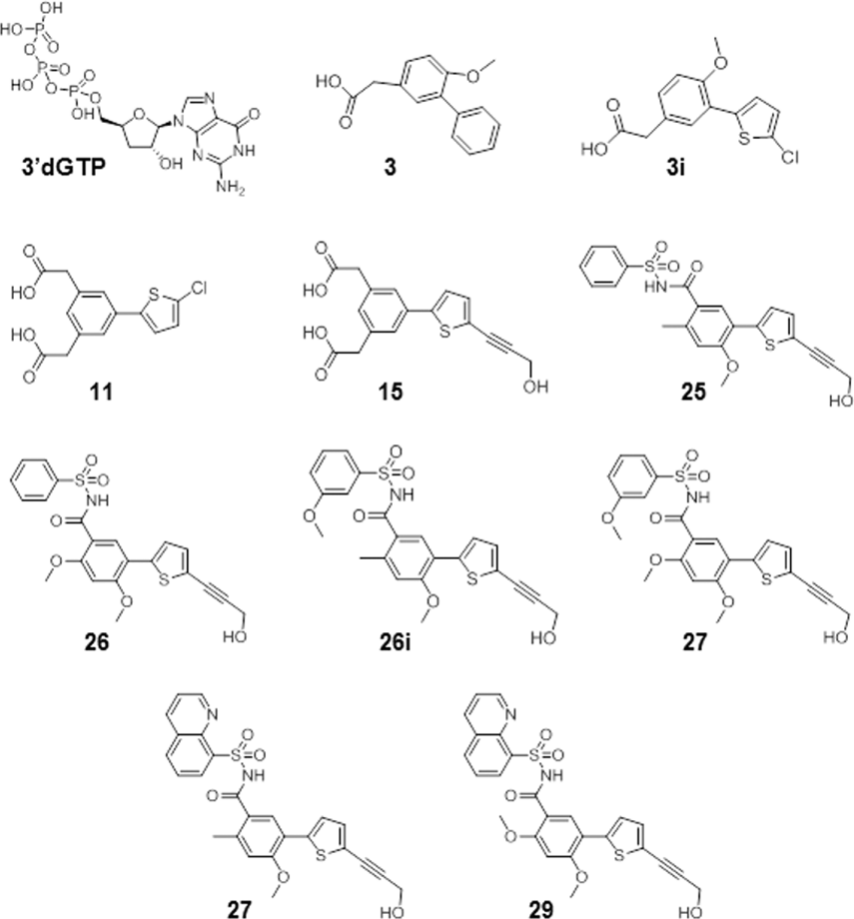
Structures of N-pocket inhibitors. N-pocket inhibitors listed in order of increasing potency in DENV enzyme and replicon cell-based assays. Compound **3** is the original hit identified from the fragment-based screening [37]. Compounds **3i** to **29i** were synthesized through rational design [38] following compound testing in DENV4 FL NS5 *dn*I assay, SPR analyses and X-ray co-crystallography.

Following fragment-based screening using X-ray crystallography, we identified **3**, a bi-phenyl acetic acid fragment, that bound to a pocket in the DENV3 RdRp thumb subdomain (IC_50_≈ 734 μM; Fig 1 and Table 1; 37). Iterative rounds of structure-guided design led to compounds that inhibited both DENV polymerase activity and viral replication in cells (Fig 1 and Table 1; Fig 1A and 1B in S1 Text). Firstly, switching the distal unsubstituted phenyl ring in **3**, with a thiophene ring (**3i**) improved compound potency by >12-fold in DENV1-4 polymerase *de novo* initiation (*dn*I) enzyme assays [38, 39]. Substitution of the methoxyl group on the outer phenyl ring with a second acid moiety increased potency in DENV-1 and -3 (compare **3i** and **11**). Replacement of the chloro-substituent on the thiophene ring in **11**, with a propargyl alcohol, markedly increased compound inhibitory property. Compound **15**, which bears this moiety, was >16-fold more active across DENV1-4 enzymes. Whilst subsequent derivatives, exemplified by compound **15,** displayed low nano-molar potencies across DENV1-4 *dn*I polymerase assays, they failed to inhibit DENV replication in cells. This is probably due to unfavorable physicochemical properties that limited their cell permeability (likely due to the presence of bis-carboxylic acid groups in **15**).

**Table 1:** Inhibitory properties of DENV polymerase N-pocket compounds.

	DENV FL NS5 de novo initiation FAPA, IC_50_ (μM)				HuH-7 DENV Replicon (μM)	
	DENV4	DENV1	DENV2	DENV3	DENV2	
Compound	IC_50_	IC_50_	IC_50_	IC_50_	EC_50_	CC_50_
3'dGTP	0.79 ± 0.20	0.52	0.62	0.27	>100	>100
**3**	733.5 ± 10.6*	768.8	768.9	796	>100	>100
**3i**	30.7 ± 9.4	46.62	37.25	66.76	>100	>100
**11**	26.0 ± 3.7*	7.1	39.74	10.7	>100	>100
**15**	1.66 ± 0.35*	0.30	2.20	0.46	>100	>100
**25**	0.165 ± 0.108*	0.033	0.106	0.040	11.4	>100
**26**	0.251 ± 0.129*	0.075	0.145	0.057	21	>100
**26i**	0.109 ± 0.061	0.024	0.086	0.054	12.1	>50
**27**	0.172 ± 0.097*	0.068*	0.106*	0.048*	3.9 ± 0.62	>100
**29**	0.023 ± 0.001*	0.013	0.038	0.016	1.9 ± 0.2	>50
**29i**	0.074 ± 0.031	0.027	0.057	0.026	2.1 ± 0.6	>50

N-pocket inhibitors listed in order of increasing potency in DENV enzyme and replicon cell-based assays. IC_50_ values from DENV *de novo* initiation FAPA assay were obtained from dose response testing of compounds (10-point, 3-fold serially diluted compounds from 0–20 μM) and are averaged from ≥3 independent experiments with DENV4 FL NS5 or from one experiment each with DENV-1, -2 and -3 FL NS5 [22]. Briefly, compounds were incubated for 20 min with enzyme alone, after which reactions were started with the ssRNA and nucleotide substrate components, and allowed to proceed for 2 hr [39]. Hill slopes for IC_50_ curves (Fig 1A in S1 Text) ranged from -0.7 to -1.6. Determination of compound inhibition (EC_50_) and cytotoxicity (CC_50_) in stable HuH-7 DENV-2 replicon cells [40] was performed 1–2 times. Cells were incubated for 48 hr in increasing compound concentration (10-point, 2-fold serially diluted compounds from 0–100 μM) after which cellular renilla luciferase (EC_50_) or ATP (CC_50_) levels measured as relative light units (RLU) were determined. All data points were measured in duplicates.

*DENV4 FL NS5 IC_50_ value for compound 3 was reported in [37, 38]. DENV4 FL NS5 IC_50_ value for compounds **11**, **15**, **25**, **26** and **29**, as well as DENV1-4 FL NS5 IC_50_ values for compound **27** were reported in [38].

Successive design strategies produced compounds with acyl-sulfonamide derivatives (replacing the charged acid groups with the acyl-sulfonamide bio-isosteres increases lipophilicity) with EC_50_ ≥ 2 μM, in a HuH-7 DENV2 replicon cell-based assay (Fig 1 and Table 1; Fig 1B in S1 Text). The most active compounds in this series, **29** and **29i**, bear the 8-quinolinol moiety, and demonstrated IC_50_ values ranging from 0.013 to 0.074 μM across DENV1-4 polymerase, with EC_50_ value of ~2 μM in the DENV2 replicon cell-based assay (Table 1).

### Kinetic studies of DENV RdRp inhibition

To better understand the inhibition mode of this class of compounds, order-of-reagent addition experiments were performed using the DENV *dn*I FAPA assay (Table 2). The standard assay format involved compounds exposed to enzyme alone followed by reaction initiations with ssRNA template and NTPs [39]. In the first experiments, compounds **15**, **27** and **29** were exposed to pre-formed enzyme-ssRNA complexes, followed by reaction initiation with NTPs. IC_50_ values generated for **15** and **27** were similar to the standard assay format, suggesting that these compounds do not discriminate between the apo-enzyme and the polymerase bound to ssRNA. Compound **29**, showed about 3-fold reduction in potency. Next, compounds were exposed to elongated enzyme-dsRNA complexes, in which the active site was occupied by the ssRNA template and newly synthesized short RNA products AGAA or AGAACC. Resulting compound inhibitory potencies dropped by 8–15 fold. The change was most pronounced in compound **29** (10–15 fold decline). These findings imply that during transition from initiation to the elongation complex, to accommodate the growing dsRNA product, the N-pocket underwent conformational changes, leading to decrease in compound binding affinities. To verify these findings, we tested the compounds in the DENV elongation FAPA assay by exposing the compounds to enzyme alone, followed by reaction initiation with duplex hetero-polymeric RNA templates [25]. Similarly, compound potencies were markedly reduced. Their IC_50_ values were 10–23 folds weaker than in the standard *dn*I assay, with **27** showing the greatest change in potency. Nevertheless, compound **29** retained potent inhibitory activity, with IC_50_ values ranging from 0.023–0.427 μM across the different enzyme assays and formats. Control 3’dGTP showed similar IC_50_ values in order-of-addition reagent experiments and in the DENV elongation FAPA assay.

**Table 2:** Inhibitory and binding properties of DENV polymerase N-pocket compounds.

Experiments with DENV4 FL NS5	Compounds	3’dGTP	15	27	29
*de novo* IC_50_ (μM); order of addition experiments [fold change]	Enzyme + compound	0.79 ± 0.20	1.66 ± 0.35*	0.172 ± 0.097*	0.023 ± 0.001*
	[Enzyme + RNA] + compound	0.44 ± 0.02	1.93 ± 0.77 [1.1X]	0.20 ± 0.07 [0.84X]	0.073 ± 0.02 [3.2X]
	[Enzyme + RNA+ATP+GTP] + compound	0.74 ± 0.26	12.9 ± 4.0 [7.8X]	2.2 ± 1.91 [9.3X]	0.338 ± 0.12 [14.7X]
	[Enzyme + RNA+ ATP+GTP+ATTO-CTP] + compound	0.71 ± 0.20	13.3 ± 2.6 [8X]	1.89 ± 1.56 [8X]	0.239 [10.4X]
Elongation IC_50_ (μM)	Enzyme + compound	0.43 ± 0.29	16.2 ± 4.7 [9.8X]	5.46 ± 2.14 [23X]	0.427 ± 0.013 [18.6X]
SPR	*K*_d, μ_M	n/a	1.44	0.0745*	0.00725*
	*K*_off, μ_M	n/a	n/a	2.38	0.642
	*K*_on, μ_M	n/a	n/a	32000000	88500000
	% R_max_		n/a	66.8	20.9
Change in protein melting temperature, Tm [+ DMSO → +50 μM compound], °C	RdRp domain	n/a	15 [50→65]	8 [50→58]	14 [50→64]
	FL NS5	n/a	9.5 [42.5→52]	4 [42.5→46.5]	9 [42.5→51.5]
	FL NS5 from BHK-21 DENV2-NGC replicon cell lysate	n/a	nd	5 [42.5→47.5]	7.5 [42.5→50]

Order-of-addition experiments were performed with DENV4 FL NS5 *de novo* initiation FAPA assay with 10-point, 3-fold serially diluted compounds from 0–20 μM, to determine effects on inhibitory properties of compound **15**, **27** and **29**, as described in Materials and Methods. Compounds were incubated for 20 min with enzyme alone, enzyme-ssRNA complex, enzyme-dsRNA complex (comprising ssRNA template and newly synthesized AG or AGC RNA products), after which reactions were started with the corresponding missing ssRNA and/or nucleotide components, and allowed to proceed for 2 hr. IC_50_ values were averaged from ≥3 independent experiments with compound **15** and **27**, and determined from at least one experiment for **29**. IC_50_ values obtained from elongation assays were averaged from >3 independent experiments for all three compounds. Hill slopes for IC_50_ curves ranged from -0.7 to -1.6. All data points were measured in duplicates. Binding affinities (*K*_d_) of compounds were determined by surface plasmon resonance using a Biacore T200 instrument as described in Materials and Methods and analyzed using Biacore T200 evalution with affinity-kinetics analysis. Effects of compounds on protein thermo-stability (melting temperature, T_m_) was assessed using the thermo-denaturation assay as described in Materials and Methods, with *in vitro* expressed recombinant DENV4 FL NS5, RdRp domain (aa 268–900), or cell lysates from BHK-21 DENV2 (strain NGC) replicon cells following the cellular thermal shift assay described previously [41]. Proteins or cell lysates were incubated with 50 μM compound or control 5% DMSO alone, followed by thermo-denaturation. Experiments were performed in duplicates.

“n/a” and “nd” respectively denote “not applicable” and “experiment not done”.

*DENV4 FL NS5 IC_50_ value for compounds **15**, **27** and **29** and *K*_d_ values for compounds **27** and **29** previously reported in [38].

**Fig 2:**
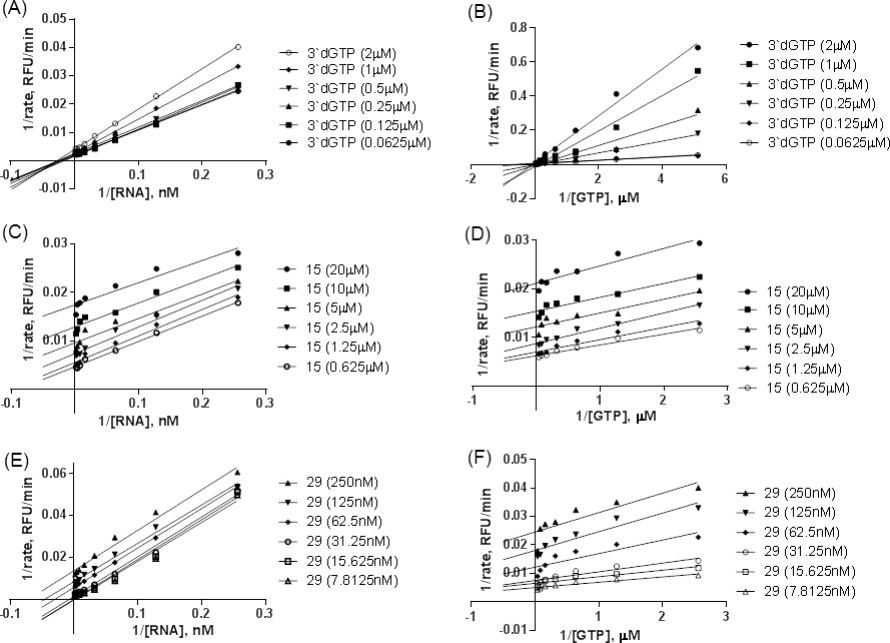
Enzyme inhibition kinetics of N-pocket compounds against DENV polymerase. DENV4 FL NS5 *dn*I FAPA assays [39] were performed in increasing concentrations of 3’dGTP control (A, B), N-pocket inhibitor, **15** (C, D) or **29** (E, F) with 0–500 nM RNA (A, C, E) or 0–50 μM GTP (B, D, F) to determine mechanisms of inhibition with respect to either substrates. Representative Lineweaver-Burk plots were derived from non-linear regression curve fitting using GraphPad Prism software. Control inhibitor 3’dGTP shows competitive inhibition with respect to GTP and non-competitive inhibition, with respect to RNA substrate.

We proceeded to characterize the inhibition kinetics of compounds **15** and **29**, in the *dn*I FAPA assay, using 3’dGTP, as a control (Fig 2; Fig 2 in S1 Text). As expected, kinetics studies using Lineweaver-Burk plots showed that 3’dGTP was a competitive inhibitor of GTP, but a non-competitive inhibitor of the viral RNA substrate. Both **15** and **29** exhibited uncompetitive inhibition profiles with respect to the viral ssRNA template. Results from kinetic competition experiments with GTP were more complex. Lineweaver-Burk plots of **15** and **29** were indicative of uncompetitive inhibition. However, at high GTP concentrations, a non-competitive mode of inhibition by these compounds was apparent. Both *de novo* initiation and elongation activities occur in the DENV polymerase *dn*I assay. For the rate-limiting *de novo* RNA synthesis step, the *K*_m_ for GTP was found to be >20 μM [19], whilst a low *K*_m_ value (0.2–0.4 μM; 18, 39) was reported for the elongation phase. It is possible that the mixed inhibition profiles for both compounds, reflect differential effects on the *dn*I and elongation phases of the enzyme activities. Indeed, the significantly weaker inhibitory capabilities of these compounds in the DENV elongation assay support this hypothesis. Furthermore, both compounds are not true un- or non-competitive inhibitors as they are also able to bind to the apo-enzyme with high affinity (see below).

### Crystal structures of DENV RdRp bound to compounds 27 and 29

**Fig 3:**
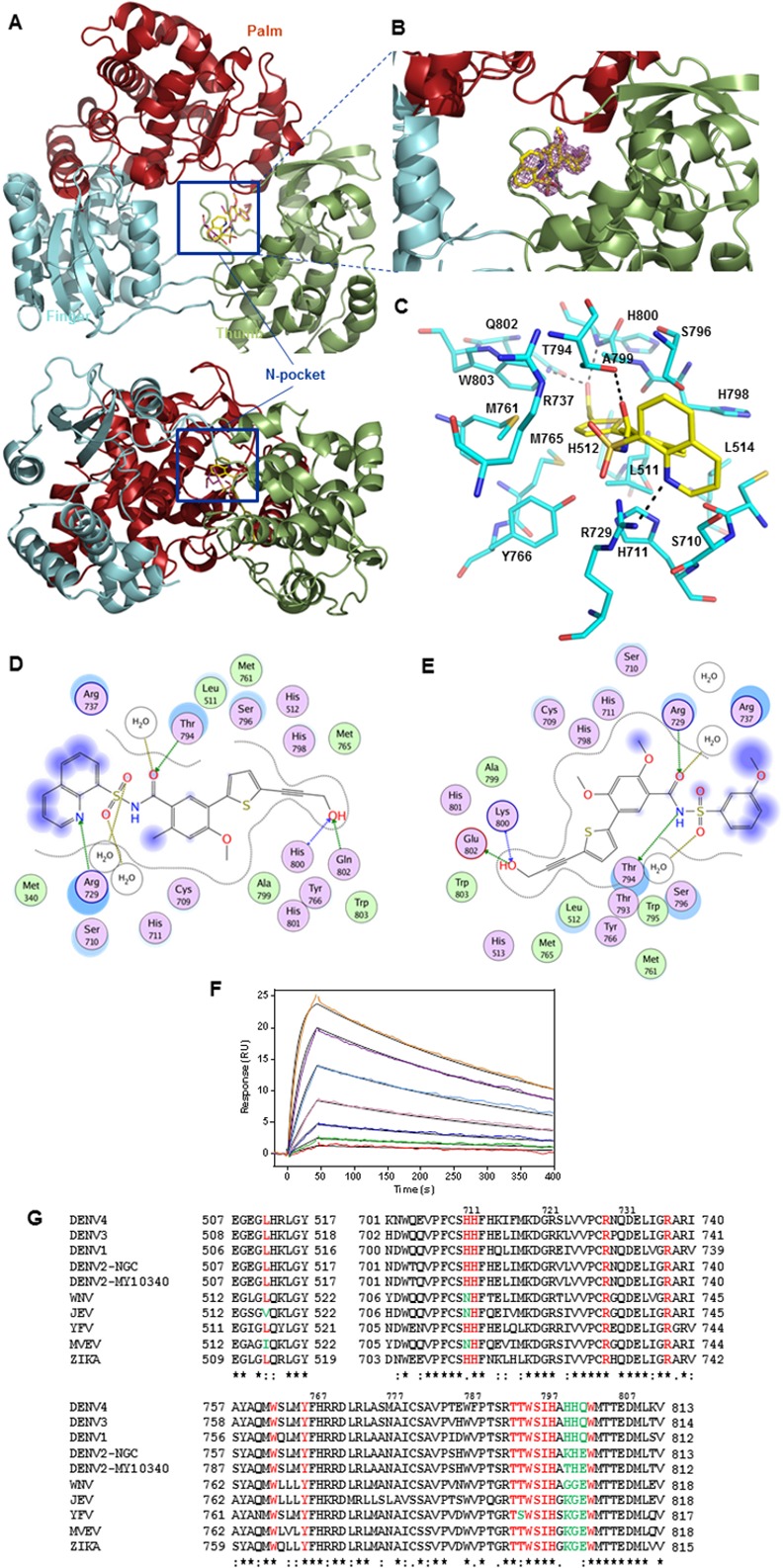
Crystal structure and binding of compounds 27 and 29 to DENV3 RdRp. (A) Overview of DENV3 RdRp bound to compound **29** (boxed), displayed as ribbons with the palm subdomain in red, finger subdomain in cyan, thumb subdomain in olive and compound **29** shown as yellow sticks. The lower left panel shows a view from “the bottom of the RdRp structure” with respect to the usual “front view”. (B) Close-up view of compound **29** binding site in the N-pocket. A difference electron density map with Fourier coefficients Fo-Fc where the compound was omitted from the phase calculation is overlaid and contoured at 3 σ level. (C) Residues lining the N-pocket for **29** are shown as cyan sticks with the compound shown as yellow sticks. Individual residues are labelled according to their numbering in the DENV3 polymerase and H-bond interactions are indicated with dashed lines. Two-dimensional ligand-interaction maps of co-crystals of DENV3 (D) and DENV2 (E) RdRp domains, bound with compounds **29** and **27**, respectively, were generated using Molecular Operating Environment. Polar residues are colored light purple, charged residues have an additional blue ring and lipophilic residues are green. The degree of solvent exposure is shown by the blue halos. H-bond interactions to the amino acid side chain or main chain are shown as dashed green or blue arrows respectively, pointing towards the H-bond acceptor. Water-mediated contacts are shown as gold dashed lines. (F) Binding of **27** to the DENV4 RdRp using surface plasmon resonance in a two-fold serial dilution from a top concentration of 2.5 μM. Raw data (coloured lines) was globally fitted to a simple 1:1 binding model (black lines). (G) Multiple alignment of amino acid residues of compound binding pocket (coloured in red and green) across different members of the Flavivirus family derived from Clustal Omega program [42]. Phylogenetic tree representing their relatedness is shown in Fig 4 in S1 Text. Strictly conserved amino acids are marked with an asterisk below the sequences (and in red font), semi-conserved residues with a colon (and in green font) whilst poorly conserved residues are marked with a dot.

DENV3 RdRp co-crystal structures with **27** and **29** solved to 2.45 Å and 1.88 Å resolution respectively (Table 3), show that the compounds occupy about 60% of the N-pocket volume and establish multiple polar contacts with several neighboring amino acid residues (Fig 3). The RdRp retains essentially the same structure as in its unbound form [21, 22] with RMSD of 0.25 Å for 612 superimposed α-carbon atoms (the RMSD is 0.18 Å between the two complexes). The compound binding mode is reminiscent of other closely-related analogs [38] with complete overlap in the positions of their most buried moieties: the thiophene ring and propargyl alcohol, whilst acyl-sulfonamide and the solvent-exposed ring: 8-quinolinol ring in **29** and methoxy-substituted phenol ring in **27**, adopt different orientations. The sulfur of the thiophene ring makes a non-covalent interaction with the side-chain hydroxyl of S796, whilst the terminal propargyl alcohol arm extends deeply into a tunnel lined by residues W803, M761, and M765. Its terminal hydroxyl group forms H-bond interactions with the backbone amide of H800 and the side-chain of Q802, and displaced a single buried water molecule present in the RdRp apo structure [21, 22]. In addition, the acyl-sulfonamide carbonyl moiety forms hydrogen bonds with the side chain of T794, and additionally in **27**, with the backbone amide of W795 via an intercalated water molecule. Co-crystallization of compounds **27** and **29** with DENV3 FL NS5 led to the same binding mode as observed for the polymerase domain (Fig 3 and Table 1 in S1 Text). Soaking of compound **27** in crystals of DENV2 (NGC strain) RdRp domain also generated the same binding mode, with the OH- moiety of the propargyl arm forming similar hydrogen bonds with residues K800 (backbone N) and E802 (carboxylic acid side chain), as H800 and Q802 in the DENV3 RdRp-**29** co-crystal structure (Fig 3D and 3E).

**Table 3:** Data collection and refinement statistics of DENV3 RdRp co-crystals.

DENV RdRp domain	DENV2	DENV3	
Compound	27	27	29
Wavelength (Å)	1.0	1.0	1.0
Resolution range (Å)	91.32–2.01	31.48–2.45 (2.54–2.45)	27.53–1.88 (1.95–1.88)
	(2.02–2.01)		
Space group	I 2 2 2	C 2 2 2_1_	C 2 2 2_1_
Unit cell (Å)	82.14 115.77	161.36 176.11 57.91	161.14 175.90 57.80
	148.59		
Measured reflections	314936 (3079)	61416 (5995)	132568 (12984)
Unique reflections	47145 (444)	30781 (3011)	66422 (6507)
Multiplicity	6.7 (6.9)	2.0 (2.0)	2.0 (2.0)
Completeness (%)	100 (100)	99.80 (99.54)	98.87 (97.92)
Mean I/sigma (I)	24.3 (3.74)	14.89 (2.25)	17.74 (2.09)
Wilson B-factor (Å^2^)	28.7	41.76	29.34
R-merge^a^	0.062 (0.617)	0.04424 (0.3635)	0.03017 (0.4225)
R-meas	0.066 (0.668)	0.06257	0.04267
CC1/2	0.997 (0.945)	0.998 (0.772)	0.999 (0.754)
CC*	-	0.999 (0.933)	1 (0.927)
R-work^b^	0.178	0.1685 (0.2352)	0.1783 (0.2995)
R-free^c^	0.216	0.2205 (0.3130)	0.2060 (0.3135)
Number of non-hydrogen atoms	5128	5177	5412
macromolecules	4640	4782	4788
Ligands	59	35	36
Water	429	360	588
Protein residues	563	585	585
RMS(bonds)	0.01	0.014	0.014
RMS(angles)	0.97	1.78	1.64
Ramachandran favoured (%)	97	96	98
Ramachandran outliers (%)	0	0.17	0.51
Clash score	1.41	2.31	1.26
Average B-factor (Å^2^)	44.9	50.00	40.20
macromolecules	45.0	49.60	38.90
Ligands	34.7	48.50	29.00
Solvent	44.6	54.40	52.10

Statistics for the highest-resolution shell are shown in parentheses.

*The numbers in parentheses refer to the last (highest) resolution shell.

^a^ R_merge_ = ∑|*I*_j_ − < *I* > |/∑*I*_j_, where *I*_j_ is the intensity of an individual reflection, and < *I* > is the average intensity of that reflection.

^b^ R_work_ = ∑||*F*_o_| − |*F*_c_||/∑|*F*_c_|, where *F*_o_ denotes the observed structure factor amplitude, and *F*_c_ the structure factor amplitude calculated from the model.

^c^ R_free_ is as for R_work_ but calculated with 5% (3044) of randomly chosen reflections omitted from the refinement.

The 10-fold higher binding affinity of **29** over **27** for DENV RdRp, observed in SPR analyses (Table 2) can be accounted for by formation of three additional hydrogen-bonds between the 8-quinolinol ring of **29** with the side chain of R729 (these favorable contacts are absent in **27** with the corresponding ring pointing towards the solvent away from R729). Thermo-denaturation studies using recombinant DENV FL NS5, RdRp and FL NS5 from DENV-replicon lysates further corroborated these findings. In these experiments, **29** consistently stabilized DENV polymerase better than **27**, leading to 2.5–6 °C better increase in protein melting temperatures compared to **27** (Table 2; Fig 5 in S1 Text). Compound binding fits to a simple 1:1 binding model in SPR analyses, correlating well with the X-ray crystallography data (Fig 3F).

### Residues lining the N-pocket play an important role in *de novo* initiation activity

To assess the functional relevance of the N-pocket for DENV polymerase activity, we targeted RdRp residues interacting with **27** or **29** as well as residues lining the N-pocket (Fig 3G), and measured both *de novo* initiation (*dn*I) and elongation activities of the corresponding RdRp Ala mutants. All mutant proteins studied have similar melting temperatures as WT, indicating that stabilities of the protein structures were not compromised by the alanine substitutions (Table 4). Overall, the results indicate that N pocket residues play an important role in DENV polymerase *dn*I and have less impact on elongation. This is particularly evident in the S710A and R737A mutants, where *dn*I activities were substantially reduced, to 26.6 and 0%, respectively, compared to WT, whilst retaining about 72% elongation activity. Both residues are completely conserved across the Flavivirus family (Fig 3G). Thus, the N pocket conformation observed in the inhibitor-bound crystal structures is likely to correspond to the structural state adopted by the DENV RdRp during *dn*I [21, 22].

**Table 4:** Alanine substitutions of N-pocket amino acid residues that interact with compounds 27 and 29.

Amino acid residues mutated to Alanine		Activity after 2 h compared to WT (%)		Protein melting temperature (°C)	Fitness after 72 hr compared to WT (%)
DENV3	DENV4	De novo initiation	Elongation		DENV4 Replicon
WT	WT	100	100	37	100
S710	S711	26.6	71.6	37	0
H711	H712	33.0	56.0	35	nd
R729	R730	33.9	37.3	37	0
R737	R738	0	72.2	38	0
Y766	Y767	55.4	85.2	38	0
T794	T795	64.1	101.9	37	30
S796	S797	65.8	78.3	37	30
H798	H799	69.5	92.9	37	nd
H800	H801	61	76.1	37	10
Q802	Q803	49.9	65.5	37	13
W803	W804	66.4	97.6	37	0

Effects of alanine mutations of pocket amino acid residues on DENV4 polymerase activity in biochemical and DENV4 replicon cell based assays. *De novo* initiation and elongation FAPA assays were performed as described previously [25, 39]. Results of average percentage *de novo* initiation and elongation activities of mutant DENV4 FL NS5 proteins, were compared against WT protein and derived from average relative fluorescence units (RFU) obtained from one experiment. All data points were measured in triplicate. WT and mutant DENV4 replicons were electroporated into BHK-21 cells and assayed for renilla luciferase activities as described in Materials and Methods. Results of percentage mutant DENV4 replicon activities were compared against WT replicon after 72 hr post-electroporation, and derived from average relative light units (RLU) obtained from one experiment.

“nd” denotes “experiment not done”.

To further validate the mode of binding of this class of inhibitors in the N-pocket, we performed inhibition assays using mutant proteins S796A and W803A, both of which retain about 66% *de novo* initiation activity (Table 5). Residue S796 interacts with the sulfur-atom in the thiophene ring whilst W803A lines the propargyl alcohol tunnel. As a control, we used 3’dGTP, which retained the same IC_50_ when tested on the mutant enzymes. All four compounds exhibited more than 10 fold increases in their IC_50_ values, when assayed with S796A and W803A mutant RdRp. Compounds **27** and **29** gave the greatest IC_50_ shifts when tested with mutant W803A (107- and 70-fold respectively). In agreement with the X-ray crystallography data, **11**, which bears a–Cl substituent instead of the extended propargyl alcohol arm on the thiophene ring, showed only 9–10 fold increase. Taken together these biochemical studies substantiate the binding modes observed in the X-ray co-crystal structures.

**Table 5:** Effect of alanine mutations of pocket amino acid residues on compound inhibitory properties on DENV4 polymerase *de novo* initiation activity.

Compound	WT IC_50_ (μM)	S797A IC_50_ (μM) [IC_50_ fold change compared to WT]	W803A IC_50_ (μM) [IC_50_ fold change compared to WT]
**11**	26.0 ± 3.7	266 [10.2]	245 [9.4]
**15**	1.66 ± 0.35	62 [37.4]	37.1 [22.4]
**27**	0.172 ± 0.10	6.22 [36.3]	18.4 [107]
**29**	0.023 ± 0.001	0.31 [13.5]	1.6 [69.6]

IC_50_ values were obtained from dose response testing of compounds (10-point, 3-fold serially diluted compounds from 0–20 μM) from one experiment with WT or mutant DENV-4 FL NS5. Briefly, compounds were incubated for 20 min with enzyme alone, after which reactions were started with the ssRNA and nucleotide substrate components, and allowed to proceed for 2 hr [39]. Hill slopes for IC_50_ curves ranged from -0.7 to -1.6. All data points were measured in duplicates.

### Reverse genetics studies of the DENV polymerase N-pocket

**Fig 4:**
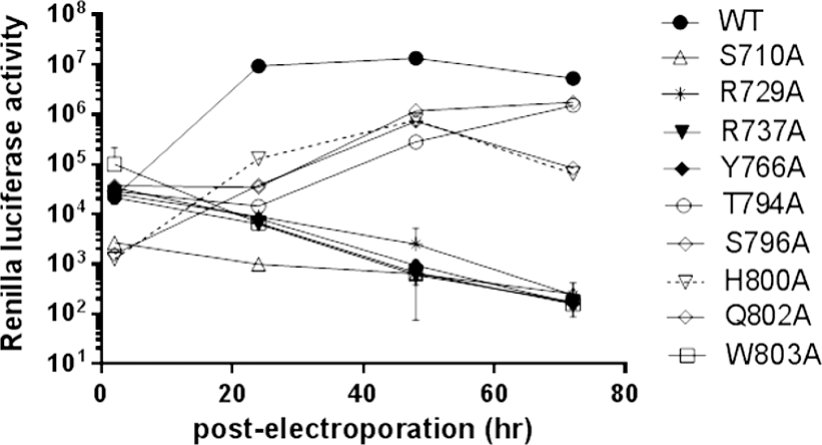
Growth kinetics of WT and N-pocket mutant DENV4 replicons. *In vitro* transcribed WT and mutant replicon cDNAs bearing single alanine substitutions in NS5 polymerase N-pocket amino acid residues were electroporated into BHK-21 cells and the levels of cellular renilla luciferase measured over three days using the renilla luciferase assay system (Promega, USA) with a Clarity luminescence microplate reader (BioTek, USA). Amino acid residues denoted DENV3 numbering (see Table 4).

We next investigated the biological effects of alanine mutation of residues S710, R729, R737, Y766, T794, S796, H800, Q803 or W803 in the context of a DENV4 replicon (Fig 4, Table 4). Following electroporation into BHK-21 cells, WT replicon replicated robustly, generating renilla luciferase signals that were 422-fold above background levels (measured at 24 hr post-electroporation). Its growth rate subsequently plateaued at 48 hr (605-fold above background). Thereafter, luciferase levels dropped 25-fold at 72 hr post-transfection. In comparison, mutant replicons bearing R729A or R737A substitutions were non-replicative, a result that is in good agreement with their poor *in vitro* NS5 polymerase activity profiles (Table 4). R729A RdRp exhibited about 30–40% of both *dn*I and elongation activities whilst *dn*I activity of R737A was completely abolished. Mutant replicons S710A, Y766A, and W803A were also non-replicative, despite showing 27–98% *de novo* initiation and elongation activities *in vitro*. Mutant replicons H800A and Q802A were poorly replicative, although their polymerase activities were only moderately decreased in the enzyme assays. In contrast, mutant replicons with T794A, S796A with similar reductions in polymerase activities, were more replicative, and continued to expand during the three-day incubation period. At 24 hr post-electroporation, their luciferase activities were respectively 838- and 456-folds poorer than WT replicon activity. By day 3, the difference had narrowed to 4.5- and 5.1-fold, respectively, lower than WT replicon activity. The reason for the difference in the replicative profiles of these latter four DENV4 mutants is uncertain. It is possible that within the context of the replicative complex, mutating these residues produced subtle differential effects on NS5 polymerase activity that translate in large variation in terms of virus replicon fitness.

### Compounds targeting the N pocket are active in DENV cell-based assays

Using DENV cell-based assays, we next examined the inhibitory properties of four potent compounds in the series (Table 6). Compounds **27**, **29** and **29i** demonstrated similar EC_50_ values ranging from 1–5 μM in DENV2 replicon cells from HuH-7, BHK-21 and A549 backgrounds while **26i** was slightly less active (EC_50_ = 12–15 μM). When inhibition studies were performed with clinical isolates of DENV1-4, **27** inhibited all four serotypes with EC_50_ ~2 μM. Compound **29i** was the next most potent compound with EC_50_ values of 1–6 μM. Despite demonstrating significantly higher potency against DENV1-4 RdRp in the *dn*I enzymatic assays, **29** displayed relatively lower cellular inhibition (EC_50_ = 4–13 μM), particularly with DENV-2 and -4 (EC_50_ = 14.1 μM and 10.2 μM respectively). Similar to the observation with replicon cells, **26i** was also the least active against DENV1-4 viruses. All four inhibitors were inactive against HCV replicon and human rhinoviruses (with EC_50_ values >25 and >50 μM. respectively). No cytotoxicity was observed in five different cell lines tested (EC_50_ >50 μM).

**Table 6:** Inhibitory profiles of N-pocket compounds in DENV cell-based assays.

Compound		26i	27	29	29i
DENV4 FL NS5 *dn*I FAPA, IC_50_ (μM)		0.109 ± 0.061	0.172 ± 0.097	0.023 ± 0.001	0.074 ± 0.031
DENV2-NGC Ren-LUC Replicon, EC_50_ (μM)	HuH-7	12.1	3.9 ± 0.6	1.9 ± 0.2	2.1 ± 0.6
	BHK21	nd	3	4.6	3.4
	A549	15.4	2.3	2.8	1.2
DENV2-NGC EGFP Replicon, EC_50_ (μM)	BHK21	nd	3.5	5.8	3.4
DENV1/2^╫^/3/4HCI, EC_50_ (μM)	A549	15.2/26.9±8.8/ 16.8/20.4±3.5	1.8±0.1/2.3±0.5/ 1.8±0.5/1.8±0.2*	6.3±0.9/14.1±3.5/ 3.8±0.7/10.2±3.4*	2.1/6.2±1.2/ 1.3±0.01/2.7±0.3
Human rhinovirus (7 serotypes), EC_50_ (μM)	HeLa	nd	>50	>50	nd
HCV replicon, EC_50_ (μM)	HuH-7	nd	>25	>25	nd
CC_50_ (μM)	HuH-7	>50	>50	>50	>50
	BHK-21	nd	>50	>50	>50
	A549	>50	>50	>50	>50
	HeLa	nd	>50	>50	nd
	HepG2	nd	>50	>50	nd

Stable HuH-7, BHK-21, and A549 DENV2 (strain NGC) replicon reporter (renilla luciferase or EGFP) cells [40], stable HuH7-HCV replicon cells [43], DENV1-4 infected A549 cells or human rhinovirus infected HeLa cells, were incubated for 48 hr in increasing compound concentration (10-point, 2-fold serially diluted compounds from 0–100 μM) after which cellular renilla or firefly luciferase (relative light units), EGFP (relative fluorescence units) or antibody-stained DENV envelope levels (relative fluorescence units, by high content imaging, HCI) were respectively measured. Compound inhibition of human rhinovirus replication in HeLa cells was measured by cytopathic inhibition over a period of 5 days. Determinations of compound inhibition (EC_50_) in cell-based assays were performed once, except for DENV1-4 replication in A549 cells (high content imaging assay), where most compounds were tested ≥2 times. Compound cytotoxicity in HuH-7, BHK-21, A549, HeLa, and HepG2 were measured 1–2 times, after compound exposure for 2 (for HuH-7, BHK-21, A549) or 4–5 (HepG2 and HeLa) days. All data points were measured in duplicates.

*Results of DENV1-4 HCI data for compounds **27** and **29** were previously reported in [38].

^╫^denotes clinical DENV2 isolate MY97-10340.

Both **27** and **29i** have an extra methoxy moiety on their central phenyl ring compared to **26i** and **29**. The inhibition results indicate that the additional methoxy group present in the former compounds may be advantageous for inhibiting infectious DENV as they are consistently more active than the cognate analogs devoid of this moiety (**26i** and **29** respectively). It is possible that this additional substituent allows for the formation of an intra-hydrogen bond with the N-atom of the sulfonamide linker facilitating better cell permeability. Notably, whilst EC_50_ values of **29i** with DENV-1, -3 and -4 were comparable with **27**, its DENV2 EC_50_ value was about two-fold lower.

### Resistant DENV2 replicons raised against compound 29 harbor mutations that map to the N-pocket

**Fig 5:**
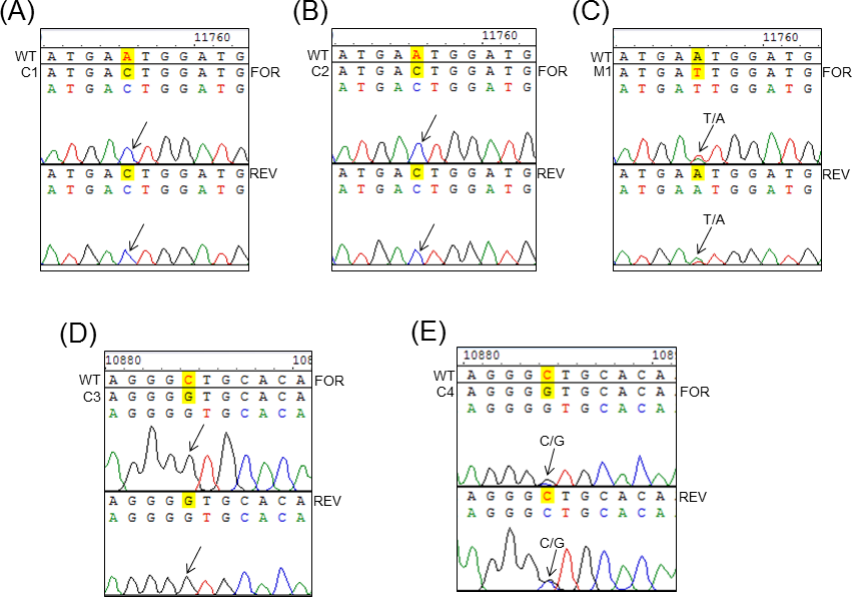
NS5 sequence analyses of compound 27- and 29-resistant DENV2 EGFP-replicon cells. (A, B) DENV2-NGC EGFP-replicon clones 1 and 2 (C1 and C2, respectively) cultured in 25 μM of compound **29** over a period of >5 weeks contained a single nucleotide change in NS5 (GAA→GAC), resulting in mutation of amino acid E802 (wildtype, WT) to D802. (C) Mixed population of DENV2-NGC EGFP-replicon cells cultured in 28 μM of compound **27**, of over a period of >6 weeks contained a single partial nucleotide change in NS5 (GAA→GAA/T), resulting in partial E802D mutation. (D) DENV2-NGC EGFP-replicon clone 3 (C3) cultured in 25 μM of compound **29** over a period of >5 weeks contained a single nucleotide change in NS5 (CTG→GTG), resulting in mutation of amino acid L511 (WT) to V511. (E) DENV2 EGFP-replicon clone 4 (C4) cultured in 25 μM of **29** over a period of >5 weeks contained a single partial nucleotide change in the same position (GTG→G/CTG), giving rise to partial L511V mutation. FOR and REV denote the nucleotide sequence information obtained with NS5 forward and reverse sequencing primers, respectively. Arrows denoted position of nucleotide change observed in replicon clones and mixed cell populations.

To confirm that the antiviral activity displayed by this series of compounds was due to the specific inhibition of RdRp, we raised resistant DENV2 EGFP-replicons using compounds **27** and **29** (Table 6). We first propagated DENV2-NGC EGFP replicon cells in 20 μM of **29** (≈1X EC_90_ value) and increased the compound concentration to 25 μM after 5 weeks. RNA was sequenced from individual colonies of resistant cells that grew in the latter compound concentration, as well as from a mixed population of cells kept in 20 μM of **29** (Fig 5). Two individual **29**-resistant replicon clones harbored the same single nucleotide change in NS5 (GAA→GAC), resulting in E802D mutation (note that residue 802 is E in DENV2-NGC and Q in DENV3 used for structure determination). A third clone contained another single nucleotide change in NS5 (CTG→GTG), resulting in L511V mutation. A fourth clone contained a mixed profile in NS5, in the same position, with both the WT nucleotide (G) as well as mutation to C nucleotide present (GTG→G/CTG), giving rise to partial L511V mutation.

Similar attempts to raise **27**-resistant cells by exposure to high concentrations of **27** (14–20 μM; ≈2X EC_90_ value) were not successful. We then exposed the DENV2-NGC replicon cells with gradually increasing concentrations of **27** (starting from 1.5 μM; ≈0.5X EC_50_). After about 6 weeks, cells in 28 μM of **27** propagated robustly, at similar rates to WT replicon cells. Further increases in compound concentrations resulted in cell death and no individual resistant colonies were obtained. RNA was sequenced from a mixed population of replicon cells maintained in 28 μM of **27**. A partial E802D mutation profile (GAA→GAA/T) was observed (Fig 5).

The crystal structure of DENV3-RdRp bound to **29** (Fig 3C) shows that the polar side chain of residue Q802 (E802 in DENV2) hydrogens bond with the hydroxyl group of the propargyl alcohol of **29**. E802D mutation results in the shortening of the amino acid side-chain by one methyl group and is likely to disrupt this H-bond formation. Residue L511 (in DENV-2 and -3) forms van der Waals interactions with the thiophene ring of **29**. In this case, loss of a methyl group in L511V is likely to weaken the interaction with the thiophene ring of the inhibitor. As a result these mutations lower the binding affinity of **29** in the N pocket. These findings thus provide compelling evidence that compounds **27** and **29** inhibit DENV replication in cells by binding to the N-pocket in the DENV polymerase.

### Impact of resistance mutations on DENV NS5 polymerase activities and compound inhibition

**Fig 6:**
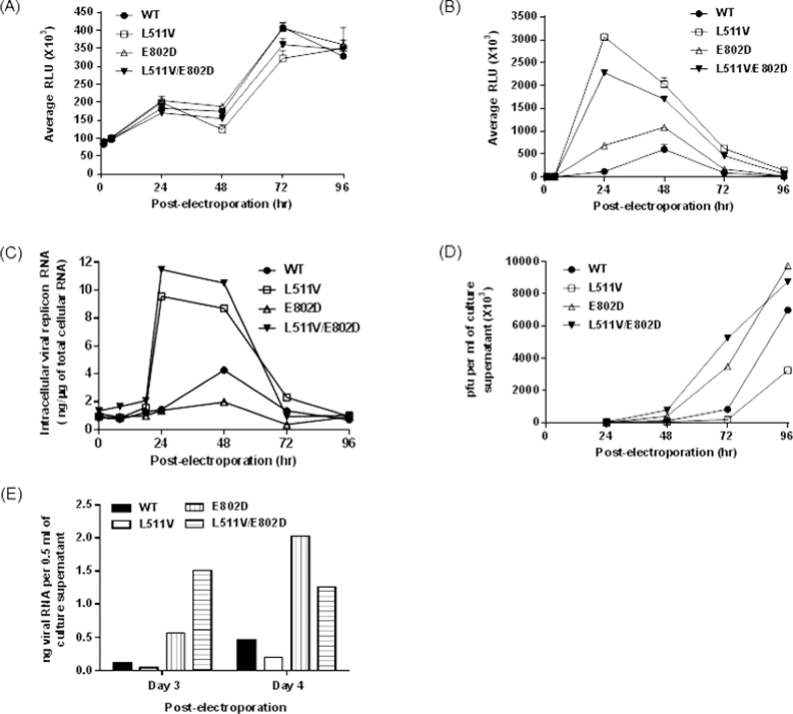
Growth kinetic profiles of DENV2 and its sub-genomic replicons with resistant phenotype amino acid changes, in absence of compound. *In vitro* transcribed WT and mutant DENV2 replicon RNA were electroporated in BHK-21 cells, after which cell viability (A) and cellular renilla luciferase levels (B) were measured over four days with the Cell-titer-Glo and renilla luciferase assay systems (Promega, USA), respectively and read with a Clarity luminescence microplate reader (BioTek, USA). (C) Intra-cellular levels of viral replicon RNA were extracted from electroporated in BHK-21 cells and measured by real-time PCR using primers for DENV2 NS5. *In vitro* transcribed WT and mutant DENV2 full length genomic RNA were electroporated in BHK-21 cells, after which culture supernatants were harvested over four days and the levels of secreted infectious virus particles were determined by plaque assay (D) or extracted for viral RNA and quantified by real-time PCR using primers for DENV2 NS5 (E). All data points were measured in duplicates.

To better understand the molecular mechanism of resistance caused by amino acid changes observed in the DENV RdRp, we generated RdRp mutants bearing these amino acid changes both in serotype DENV2 (L511V and E802D) and DENV4 (L512V and Q803N). Both the single and double mutant NS5 proteins have similar thermo-stability as WT protein and comparable *dn*I and elongation activities (Figs 6 and 7 in S1 Text). We then examined the impact of these mutations on the inhibitory capabilities of compounds **27** and **29** (Table 7). Both compounds were significantly less active against mutant enzymes than WT protein, in the *dn*I FAPA assay: IC_50_ value of **29** declined by 4–12 fold in DENV2 and DENV4, single mutants, whilst potency was further reduced in double mutant enzymes, by 52–133 fold lower than WT enzyme. For compound **27**, IC_50_ values dropped by 5-88-fold in DENV2 single and double mutants. Changes in potency against DENV4 single and double mutants were even more severe: a complete loss of inhibitory activity (IC_50_ >20 μM) was observed. Furthermore, these compounds were less effective in stabilizing the mutant enzymes compared to the corresponding WT proteins (Fig 7 in S1 Text). Taken together, both *in vitro* enzyme profiling and thermo-denaturation studies strongly corroborate the resistant replicon phenotype obtained with **29**.

**Table 7:** Inhibitory profiles of N-pocket compounds in DENV polymerase with resistant phenotype amino acid changes.

		IC_50_ (fold change compared to WT), μM	
FL NS5		29	27
DENV2-NGC	WT	0.036	0.173
	L511V	0.352 (9.9X)	2.548 (14.7X)
	E802D	0.148 (4.2X)	0.936 (5.4X)
	L511V/E802D	1.85 (52X)	15.21 (88X)
DENV4	WT	0.033	0.134
	L512V	0.28 (8.7X)	>20 (>100X)
	Q803N	0.38 (11.7X)	>20 (>100X)
	L512V/Q803N	4.37 (133X)	>20 (>100X)

IC_50_ values from DENV2 and DENV4 *de novo* initiation FAPA assays were obtained from dose response testing of compounds, **27** and **29** (10-point, 3-fold serially diluted compounds from 0–20 μM), from one experiment with WT and mutant enzymes. Briefly, compounds were incubated for 20 min with enzyme alone, after which reactions were started with the ssRNA and nucleotide substrate components, and allowed to proceed for 2 hr [39]. Hill slopes for IC_50_ curves ranged from -0.7 to -1.6. All data points were measured in duplicates.

### Impact of resistance mutations on DENV replication and compound inhibition

To evaluate the impact of L511V and E802D mutations on DENV replication, we introduced single (L511V or E802D) and double (L511V/E802D) amino acid changes into the DENV2 (strain NGC) replicon and its infectious full length virus genome. After electroporation into BHK-21 cells, replications of replicons (measured by renilla luciferase activity) or virus (measured by plaque assays) in the absence of compound, were monitored for 4 days (Fig 6; Table A in S1 Text). Electroporated cells harboring WT and mutant replicons showed similar multiplication rates and viability (Fig 6A). Compared to WT DENV2 replicon, all three mutant replicons replicated faster and generated higher levels of renilla luciferase signals (Fig 6B) as well as viral RNA (Fig 6C; Fig 8A in S1 Text) and NS5 levels (Fig 8A in S1 Text). Luciferase levels peaked at day 1 for mutants L511V and L511V/E802D, and at day 2 for WT and mutant E802D.

Experiments conducted with infectious WT and mutant DENV2 showed a different profile. Viral titers increased steadily from days 1–4 post-electroporation, unlike replicon growth curves (Fig 6D and 6E; Table 2B in S1 Text). L511V mutant produced the least infectious virus particles, compared to the other three viruses. Immunofluorescence staining of intracellular viral RNA and NS5 also revealed highest NS5 and dsRNA levels in mutant L511V (Fig 8B in S1 Text). The reason for the difference between extra- and intra-cellular viral RNA levels of mutants L511V is unclear. It is possible that mutation of this residue may have different impact on the replicon and virus.

Next, we examined the inhibitory effects of compounds **27** and **29** on the DENV2 single and double mutant replicons and viruses (Table 8). EC_50_ value of **29** was reduced by 3-6-folds in single and double mutant DENV2 replicons, compared to WT replicon. Similarly, its potency was also reduced by 5-6-folds in virus mutants compared against WT virus. These data further verify that the anti-DENV properties of **29** function through binding to the N-pocket in NS5 polymerase in DENV-infected cells.

**Table 8:** Inhibitory profiles of N-pocket compounds in DENV replicons and virus with resistant phenotype amino acid changes.

	EC_50_ (μM) (fold increase compared to WT)			
	Replicon		Virus	
DENV2-NGC	27	29	27	29
Wild Type	7.83	6.70	0.87	1.11
L512V	25.86 (3.3X)	32.32 (4.8X)	2.2 (2.5X)	6 (5.4X)
E802D	12.77 (1.6X)	21.39 (3.2X)	1.42 (1.6X)	5.8 (5.2X)
L512V/E802D	28.42 (3.6X)	38.76 (5.8X)	2.32 (2.6X)	7.01 (6.4X)
Control DENV2-NGC EGFP-replicon cells passaged in DMSO	2.46	3.55	nd	nd
Compound **29**-resistant DENV2-NGC EGFP-replicon cells	41.7 (17X)	34 (9.6X)	nd	nd

WT and mutant DENV2 (strain NGC) replicon cDNAs were electroporated in BHK-21 cells, after which cells were seeded into 96-well plates and treated with compounds, **27** and **29** (10-point, 2-fold serially diluted compounds from 0–100 μM), for 2 days. EC_50_ values from replicon cells were determined by measuring cellular renilla luciferase levels. Compound testing in WT and mutant DENV2 were performed with supernatant harvested from BHK-21, three days after electroporation of their respective viral genomic cDNAs. Virus titers were quantified by plaque assay in BHK-21 cells and used to infect A549 cells (MOI = 0.2) seeded into 96-well plates. Compounds **27** and **29** (10-point, 3-fold serially diluted compounds from 0–50 μM), were added for 48 hr, after which culture media was harvested and virus titers present were determined by plaque assay using BHK-21 cells. For determination of compound EC_50_ values in mixed populations of **27**-resistant or DMSO-cultured DENV2 replicon cells, cells were seeded into 96-well plates and treated with compounds, **27** and **29** (10-point, 2-fold serially diluted compounds from 0–100 μM), for 2 days. EC_50_ values from replicon cells were determined by measuring cellular renilla luciferase levels. All data points were measured in duplicates. nd = not done.

Potency reduction of compound **27** was less pronounced. Its EC_50_ values were reduced by 2-4-folds in mutant DENV2 replicons and viruses. The observed weaker EC_50_ shifts for **27** are puzzling as its binding mode is similar to **29** and involves non-covalent interaction of the thiophene ring with L511 and H-bond formation between the propargyl alcohol and E802D (Fig 3). Additional **27**-resistant DENV2 EGFP-replicons were raised and studied. EC_50_ values of compounds **27** and **29** shifted by 17- and 10-folds, respectively, in these cells, compared to control cells raised in DMSO. Full replicon genome sequence analyses revealed secondary mutations present in NS5 methyl-transferase and NS4B in the **27**-resistant replicon cells. Further reverse genetics on DENV2 with these amino acids are ongoing to better understand their roles in overcoming **27**-mediated DENV2 growth inhibition.

## Discussion

In this report, we characterized a novel allosteric pocket at the interface of the thumb and palm subdomains of DENV RdRp [21, 22]. This binding site, which we termed the “N pocket”, was found through a fragment-based screening approach, by X-ray crystallography, using the DENV3 apo-RdRp protein as a target [37]. It is located near the priming loop (aa782-809) of the enzyme and is lined by residues highly conserved across DENV1-4, as well as in other flaviviruses including ZIKV (Fig 3G). Alanine substitutions, demonstrated that several N-pocket residues are important for NS5 polymerase *de novo* initiation activity and also for virus replication. Accordingly, N-pocket inhibitors generated by rational design potently inhibited DENV1-4 polymerase *de novo* initiation activities and virus replication in various cell types. They bind with strong affinity to recombinant apo-enzyme as well as FL NS5 from DENV replicon cell lysates.

Compound **29,** one of the most potent compounds in the series, binds DENV RdRp with single-digit nano-molar affinity and stabilizes the RdRp melting temperature by 7.5–14 °C. It inhibits *de novo* initiation activity of DENV1-4 polymerases with IC_50_ values ranging from 13 to 38 nM. Alanine substitutions of N-pocket residues diminished the inhibitory properties of this class of compounds. Resistant DENV raised against compound **29**, harbored amino acid mutations (L511V and E802D; DENV2 numbering) that mapped to the N-pocket. Correspondingly, these amino acid alterations reduced compound potencies in DENV cell-based and RdRp enzyme assays.

Residue L511 is conserved across DENV1-4, WNV and YFV. Residues 800 (H) and 802 (Q) are conserved across DENV-1, -3 and -4 but not in DENV2 (Fig 3G). In the laboratory adapted DENV2 strain, NGC, these residues are respectively, K and E, whilst in the DENV2 clinical isolate, MY097-10340, they are T and E. Crystal structure of DENV2-NGC RdRp bound with **27** showed that the OH- moiety of the propargyl alcohol arm, made similar hydrogen bonds with residues K800 (backbone N) and E802 (carboxylic acid side chain), as H800 and Q802 in the DENV3 RdRp-**27** co-crystal structure. Residue T800 in DENV2, MY097-10340, would also be expected to form the same interaction as H800 or K800.

Kinetic studies showed that N pocket inhibitors have a mixed inhibition profile in the *de novo* initiation assay. Competitive experiments performed with GTP suggest differential inhibitory modes (non- and un-competitive) during initiation and elongation phases. Indeed, whilst compounds such as **27** and **29** have nano-molar IC_50_ values in the *de novo* initiation assay, their inhibitory potencies drop dramatically by 10–23 fold in the elongation assay (IC_50_ = 5.5 and 0.43 μM respectively). Given that both *de novo* initiation and elongation events occur simultaneously in DENV-infected cells, we speculate that N pocket inhibitors block the first activity better than the second, giving rise to the observed DENV cell-based EC_50_ values (example **29**; EC_50_ = 2–14 μM). Order-of-reagent addition experiments further corroborate this hypothesis. Compound potencies are reduced only when the enzyme is occupied with newly synthesized duplex RNA, and not by single-stranded viral RNA. Presumably, retraction of the priming loop (aa782-809) from the active site during enzyme elongation alters the conformation of the N-pocket, leading to weaker binding affinities of the RdRp for the compounds.

DENV RdRp N-pocket compounds discovered here, share some common features with Site III non-nucleoside inhibitors described earlier for the HCV polymerase [29]. Site II (thumb 1), III (palm 1) and IV (palm 2) HCV RdRp inhibitors are *de novo* initiation inhibitors that lock the thumb subdomain in a conformation that prevents *de novo* initiation. Several such HCV inhibitors possess sub-micromolar EC_50_ values in HCV replicon cell-based assays and progressed into late phase clinical trials [44]. Of note, Dasabuvir (ABT 333), a Site III inhibitor, has recently been approved for HCV therapy in combination with NS3/4A protease and NS5 inhibitors (http://hepatitiscnewdrugs.blogspot.sg/2015/07/fda-hepatitis-update-approval-of_24.html). HCV RdRp site III inhibitors bind at the interface of the thumb and palm subdomains, with one side comprising the “primer grip” and the opposite side formed by the β-hairpin loop from the thumb (equivalent to the priming loop in DENV RdRp). Inhibitor binding is promoted by interactions with both sides, in particular, with Y448 from the β-loop. The initiating nucleotide GTP, also binds to this site and forms key interactions with R386, S387 and R394 from HCV NS5B.

DENV RdRp N-pocket inhibitors also form several hydrogen bonds with residues from the priming loop that project from the thumb domain (aa794-802). Additionally, residues S710, R729 and R737 collectively form the mouth of the N-pocket and interact with the acyl-sulfonamide group from this class of inhibitors. This region of the binding pocket corresponds to the proposed *i*-1 site observed in other Flavivirus RdRps such as HCV, BVDV (discussed in [45]). The terminal aromatic rings of **27** and **29** protrude from the enzyme and are solvent-exposed. In the context of the replication complex, these compounds may differentially affect the interactions of NS5 with other viral or host proteins and further contribute to or contravene viral inhibition. Residues R729 and R737 in DENV RdRp are likely to play similar roles as R386 and R394 in HCV RdRp. They interact respectively with the γ- and β-phosphate of the GTP moiety bound in the DENV [21] and JEV RdRp active sites (corresponding to R734, R742, JEV numbering; [46]). Thus, N-pocket inhibitors could affect *de novo* initiation by interfering with the binding of the incoming +1 rNTP substrate.

Some differences with HCV NS5B are noteworthy: there is no equivalent of the HCV RdRp primer grip wall for DENV N-pocket. In addition, unlike HCV RdRp where the C-terminal loop penetrates the active site and participates in enzyme activity, the C-terminal end of flavivirus RdRp is disordered in most reported crystal structures. Interestingly, this segment was recently observed to interact with a neighbouring MTase domain in a DENV FL NS5 oligomeric structure [47]. We speculate that the absence of both regions in DENV RdRp active site, prevents formation of additional contacts with N-pocket inhibitors, and is the reason for the weaker binding affinities of N-pocket compounds, compared to HCV site III inhibitors. Design strategies that capture and order the C-terminal sequence of DENV RdRp or its G-loop [45], would likely further enhance inhibitor binding affinity and block *de novo* initiation.

Residue H798 in DENV priming loop was proposed to be the counterpart of Y448 in HCV RdRp and to be responsible for ATP-specific initiation [48]. Unfortunately, H798 is too distant to make contact with the acyl-sulfonamide moiety of the compounds and design strategies in this direction were not fruitful. However, given that the N-pocket is close to the enzyme active site, extensions of inhibitor towards the GDD motif may strengthen the compound affinity. High clearance was observed for acyl-sulfonamide propargyl alcohol compounds *in vivo* which rendered them unsuitable for mouse efficacy studies. Both the thiophene ring and the primary propargyl alcohol have potential metabolic liabilities *in vivo*. To develop N-pocket inhibitors with better pharmaco-kinetic properties, both groups would need to be replaced with more stable moieties, whilst retaining key hydrogen bond interactions, with residues such as 800 and 802.

Finally, compounds **27** and **29** were inactive when tested on the WNV replicon cell-based assay (Fig 9 in S1 Text). Previous comparisons revealed that the WNV RdRp priming loop is closer to the *i*-1 site, and prevents formation of a similar N-pocket [45]. In addition, whilst DENV N-pocket residues are mostly conserved across the flavivirus family, residues 799–802, which accommodate the propargyl alcohol arm, are more divergent (Fig 3G). Taken together, these may be the reasons for the lack of compound activity in WNV. Interestingly, residues 799–802 are more similar amongst JEV, MVEV WNV, YFV and ZIKV, compared to DENV1-4. In this light, it may not be plausible to develop pan-active N-pocket inhibitors that work on all flaviviruses. Rather, designing N-pocket inhibitors that specifically target different subgroups of the flavivirus family may be a more attainable goal.

## Methods

Methods used in this study are briefly summarized below. Full descriptions are given in Supporting Information.

### Cells, compounds, and antibodies

A549 cells (human alveolar epithelial cells; American Type Culture Collection (ATCC), USA) were maintained in Ham’s F-12K medium (LifeTech, USA) containing 10% fetal bovine serum (FBS), 1mM L-glutamine and 1% penicillin-streptomycin. BHK-21 cells (baby hamster kidney cells; ATCC, USA) were cultured in Dulbecco modified Eagle medium (DMEM; LifeTech, USA) supplemented with 10% FBS, 1 mM L-glutamine and 1% penicillin-streptomycin. C6/36 mosquito cells (ATCC, USA) were grown in RPMI 1640 medium containing 10% FBS, 1 mM L-glutamine and 1% penicillin-streptomycin. A549, BHK-21 and HuH7 (Japan Collection of Research Bioresources Cell Bank, Japan) cells containing a DENV2 (New Guinea C) renilla luciferase or EGFP sub-genomic replicon were maintained in F-12 and DMEM medium, respectively, containing 10% FBS, 1 mM L-glutamine 20 μg/ml puromycin, and 1% penicillin-streptomycin [40]. Huh-7.5 cells containing a firefly luciferase sub-genomic replicon of hepatitis C virus (HCV) genotype 1b were licensed from Apath LLC (St. Louis, MO, USA; 42) and were maintained in DMEM containing 10% FBS, 1 mM L-glutamine, 0.25 mg/ml Geneticin, and 1% penicillin-streptomycin. A549, BHK-21, DENV2 replicon, and HCV replicon cell lines were incubated at 37°C. C6/36 cells were cultured at 28°C. All compounds were synthesized in-house. DENV-specific mouse monoclonal antibody 4G2 against the DENV envelope (E) protein was prepared from a hybridoma cell line purchased from the ATCC (USA) and rabbit poly-clonal antibody against DENV2 NS5 was purchased from GeneTex (USA). Synthesis of N-pocket inhibitors are described in [38].

### Cloning, expression and activity tests of DENV1-4 WT and mutant FL NS5 proteins

Site-directed alanine mutations of DENV4 FL NS5 cDNA were performed using pET28-D4-MY01-22713 NS5FL [23] as a template, according to the manufacturer’s protocol (Stratagene, USA). Protein expressions of DENV1-4 FL NS5 and their stability analyses by thermo-fluorescence were performed as described previously [23]. DENV *de novo* initiation and elongation FAPA assays were earlier described [25].

### Crystallization and X-ray structure determination

DENV3 RdRp protein expression and crystallization was as described previously [22]. Briefly, DENV3 RdRp at 12 mg/ml was mixed with 1 mM compound **27** or **29** (prepared from a 10 mM DMSO stock to give a final concentration of 10% DMSO) prior to setting up hanging-drop vapor-diffusion crystallization trials in 0.1 M Tris/HCl, pH 8.0 and 25% PEG 500 MME. DENV2 RdRp (Strain NGC, aa 266–900) protein expression was as described previously (23). Protein crystallization was performed at 8 mg/ml in a sitting-drop vapor-diffusion setup with a well solution of 0.1 M MES pH 7.0, 0.35 mM MgCl_2_ and 16% PEG 4000 with a drop ratio of 2:1 (protein:well). Crystals appeared in one day and were transferred to the well solution supplemented with 10 mM compound **27** (prepared from a 100 mM DMSO stock to obtain a final concentration of 10% DMSO) for overnight incubation. For cryo-protection, crystals were transferred to the crystallization solution supplemented with 10% glycerol and 10% compound/DMSO and cooled in liquid nitrogen. Diffraction data were integrated using autoPROC (DENV2) or XDS (DENV3) and scaled using SCALA or AIMLESS, both part of the CCP4 suite [49]. The structures were directly refined using BUSTER, part of the global phasing suite.

### Surface plasmon resonance binding assay

Biotinylated DENV3 and DENV4 RdRp were captured on flow cells 2 and 4 respectively in 50 mM Tris/HCl, pH 7.5, 200 mM NaCl, 2 mM DTT, 0.05% Tween 20, and 3% DMSO at 4°C. Flow cell 1 and 3 were left blank to serve as a reference [37]. Compounds were tested in a 7-point 2-fold serial dilution, from 2.5 μM and a zero-concentration sample was subtracted from each run. Compounds were injected at a flow rate of 30 μL/min, with 45 s contact time and 600 s dissociation, starting from the DMSO control and finishing with the highest concentration. The experiments were performed using a Biacore T200 instrument and the data were analyzed using Biacore T200 Evaluation software, version 2.0.

### Compound testing with DENV *dn*I FAPA assays

DENV1-4 FL NS5 *dn*I assays were performed as described previously [39]. Briefly, compounds from 0–20 or -100 μM concentrations are two-fold serially diluted into 384-well black opaque plates (Corning Costar), after which 100 nM DENV FL NS5 protein was added and the plates incubated at RT for 20 min. RNA and ATTO-CTP, ATP, GTP and UTP were then added and the plates incubated for another 120 min. Reactions were stopped with buffer containing 25 nM CIP, re- incubated at RT for 60 min and read on a Tecan Safire II microplate reader. For order-of-addition experiments, DENV4 FL NS5 was incubated for one hour at RT with RNA, ATP, and GTP or RNA, ATP, GTP and ATTO-CTP, followed by exposure to serially diluted compounds for 20 min at RT. The missing components (ATTO-CTP and UTP or UTP alone) were added and the reactions continued for 120 min after which STOP buffer was added as before. All datapoints were performed in duplicate wells. Each compound was tested at least twice.

### Selection and sequencing of resistant virus

BHK-21 DENV2 (strain New Guinea C) EGFP-replicon cells [40] resistant to compounds **27** and **29** were first obtained by serial passaging of the cells in 14 μM of **27** or 20 μM of **29 (**1–2× EC_90_ values). Briefly, 1 X 10^5^ cells were seeded over-night into 6-well plates, followed by addition of fresh media containing 2% FCS and compounds. Media was changed every 2–3 day. After 5 weeks, concentration of **29** was increased to 25 μM. Individual colonies or mixed populations of resistant cells were isolated, expanded and total cellular RNA extracted. Alternatively, native replicon cells were incubated with media containing 1.5 μM of **27** (0.5X EC_50_ value) and after 3 days, fresh media with 2-fold increase in compound concentration was added. The process was repeated until cells were exposed to 28 μM **27**, after which total cellular RNA was extracted. Viral RNA was extracted by using QIAamp viral RNA minikit (Qiagen) and NS5 cDNA was amplified by SuperScript One-Step reverse transcription (RT)-PCR with Platinum *Taq* (Invitrogen) and subjected to DNA sequencing. Control cells were passaged in the presence of 0.5% DMSO.

### Generation of DENV4 NS5 mutant replicons

Mutations in the DENV4 NS5 (GenBank accession number AF326825) sequence were engineered into the subclone, pACYC-DENV4-F shuttle, using the QuikChange II XL site-directed mutagenesis kit according to the manufacturer’s protocol (Stratagene). This plasmid harbours nucleotides 7564–10653 (from NS3-3’UTR) from the DENV4, MY01-22713 strain, linked at the 3’end to the Hepatitis D virus ribozyme (HDVr) sequence. Following sequence verification, the plasmids were digested with NotI and KpnI and inserted with a PCR product comprising the sequence comprising nucleotides 1–7563 downstream of the T7 promoter in which the region from nucleotides 217–2291 in this cDNA has been replaced by renilla luciferase and foot-and-mouth disease virus 2A protease cDNAs [50].

### Construction of mutant DENV2 replicons and full-length infectious clone

DENV2 (strain New Guinea C, NGC) replicons or full-length cDNA clones with NS5 mutations were constructed with a pACYC-NGC-RLuc replicon or pACYC-NGC FL, respectively and a TA-NGC (shuttle E) vector as previously described [50]. The pACYC-NGC FL plasmid contains the T7 promoter, the DENV2 NGC genome, and HDVr. The pACYC- NGC-RLuc replicon plasmid contains the same cDNA as pACYC-NGC FL except that cDNAs encoding structural proteins were replaced by renilla luciferase cDNA [40]. The shuttle E vector contains nucleotides 5427 to 10955 (from NS3 to 3’UTR and HDVr sequence). All NS5 mutations were engineered into the shuttle E vector, using QuikChange II XL site-directed mutagenesis kit (Stratagene) according to the manufacturer’s protocol. The mutants were cloned into pACYC-NGC replicon or FL plasmid at *BspEI* and *MluI* restriction sites. All constructs were verified by DNA sequencing.

### Compound testing in DENV replicons and infectious virus and HCV replicon

A549, BHK-21 and Huh7 cells bearing stable DENV2 sub-genomic replicon [40] or transiently electroporated DENV2-NGC replicon or infectious virus cDNAs were seeded into 384-well microplate (3,000 cells per well). After over-night incubation at 37°C with 5% CO_2_, the cells were treated with 2-fold serially diluted compounds, starting from 20 or 50 μM. At 48 hr of post-incubation, renilla luciferase activities were measured with the ViviRen live-cell substrate (Promega, USA) according to the manufacturer’s protocol. CellTiter-Glo reagent (Promega, USA) was then added to determine cytotoxic effects of compounds. For the HCV replicon assay, Huh-7.5 cells harboring the HCV replicon [43] were seeded into a 96-well microplate (20,000 cells per well). At 48 hr after compound treatment, cells were assayed for firefly luciferase activity by using a Bright-Glo luciferase assay (Promega, USA). NITD-008, a nucleoside inhibitor of DENV and HCV was added as a control [33]. Compounds were tested up to 25 μM in HCV replicon cells due to limits in DMSO tolerability of these cells, and up to 50 μM in other cell types, based on compound solubility.

## Supporting Information

S1 Text **Fig 1A.** Representative IC_50_ curves for N-pocket inhibitors tested in DENV4 FL NS5 de novo initiation FAPA assay [3]. Briefly, compounds (10-point, 3-fold serially diluted compounds from 0–20 or 100 μM) were incubated at RT for 20 min with enzyme alone, in 384-well plate opaque plates, after which reactions were started with addition of ssRNA and nucleotide substrate components, and allowed to proceed for 2 hr. Reactions were stopped by addition of 10 μl of 2.5X STOP buffer with 25 nM CIP, re- incubated at RT for 60 min and read on a Tecan Safire II microplate reader (excitation_max_ and emission_max_ wavelengths 422 nm and 566 nm). Data was fitted to the four parameter logistic equation and IC_50_ curves plotted using Graphpad^®^ Prism software. Table contains average IC_50_ values and hill slopes obtained. All data points were measured in duplicates. **Fig 1B.** Representative EC_50_ and CC_50_ curves for N-pocket inhibitors tested in HuH-7 DENV-2 replicon cell-based assay [1]. Cells were seeded over-night in white opaque 384-well plates followed by incubation with increasing compound concentrations (10-point, 2-fold serially diluted compounds from 0–50 μM) for 48 hr, after which cellular renilla luciferase (EC_50_) or ATP (CC_50_) levels, measured as relative light units (RLU), were determined using ViviRen and Cell Titer-Glo (Promega) according to manufacturer’s protocol. All data points were measured in duplicates. **Fig 2.** Enzyme inhibition kinetics of N-pocket compounds against DENV polymerase. DENV4 FL NS5 *dn*I FAPA assays [5] were performed in increasing concentrations of N-pocket inhibitor, **15** or **29** or 3’dGTP (control) with 0–500 nM RNA or 0–50 μM GTP to determine mechanism of inhibition with respect to either suibstrates. Representative Michaelis-Menton plots were derived from non-linear regression curve fitting using Graphpad Prism software. **Fig 3.** Crystal structures of DENV3 FL NS5 were obtained by co-crystallization according to conditions from reference 7 as described in Supplementary Materials. (A) Overall view of the DENV3 FL NS5 structure displayed as ribbon with the methyltransferase domain in red, the linker region in orange, palm, thumb and fingers subdomains colored in olive, green and blue respectively. Both compounds 23 (magenta sticks) and 29 (yellow sticks) are overlaid in the polymerase domain. Magnified views of compound 29 (B) and 27 (C) with Fo-Fc contoured at 4 σ where each compound was omitted from the phase calculation. (D) Superimposition of the RdRp domain from FL NS5 (green ribbon) and RdRp (pink ribbon) with bound compounds 29 and 23 in sticks showing the absence of conformational changes. (E) Superimposition of compound 29 bound to FL NS5 structure (magenta sticks) and bound to RdRp structure (pink sticks) and compound 23 (panel F) bound to FL NS5 (grey sticks) and bound to RdRp (orange sticks). The compound conformations are closely superimposable. PDB codes for the FL NS5 structures with compounds 27 and 29 are 5JJS and 5JJR, respectively. **Fig 4.** Phylogenetic tree representing relatedness of N-pocket amino acid residues from different members of the Flavivirus family, derived from Clustal Omega program [8]. **Fig 5.** Effects of N-pocket compounds on DENV polymerase thermo-stability. Melting temperature (T_m_) was assessed by thermo-denaturation in presence of the SYPRO Orange dye as described in Materials and Methods. (A-F) Representative melting curves of DENV4 RdRp domain (aa 266–900; A-C) and FL NS5 (D-F) in presence of 50 μM N-pocket inhibitors or 5% DMSO control. (G) Cellular thermal shifts assays [9] were performed with BHK-21 DENV2-NGC replicon cell lysates and 40 mM of **27** or **29**. Briefly, lysates were incubated with compounds or 5% DMSO for 1 hr at 4 °C, followed by heating at 30–70 °C. Samples were spun and the supernatants loaded onto 12% SDS-PAGE gels, followed by gel electrophoresis and western blotting with anti-DENV2 NS5 antibody (GeneTex, USA). Table shows the changes in protein melting temperatures in presence of compounds compared to controls treated with DMSO. **Fig 6.** Activity profiles of DENV polymerase bearing resistant phenotype amino acid changes. Recombinant DENV2 (A, C) and DENV4 (B, D) FL NS5 proteins bearing single or double amino acid changes in the N-pocket were tested in *de novo* initiation (A, B) and elongation (C, D) FAPA assays and compared against activities of WT DENV2 or DENV4 FL NS5 proteins. Reactions were conducted over 2 hr at RT and from average relative fluorescence units (RFU) obtained from one experiment. All data points were measured in triplicate. **Fig 7.** Effects of compounds on DENV polymerase thermo-stability. Melting temperature (T_m_) was assessed by thermo-denaturation in presence of the SYPRO Orange dye as described in Materials and Methods. (A-D) Representative melting curves of *in vitro* expressed recombinant DENV2 FL NS5 WT or mutant proteins in presence of 50 μM compound or 5% DMSO control. Table shows the melting temperatures of DENV-2 and -4 FL NS5 WT and mutant proteins as well as the changes in their melting temperatures in presence of compounds compared to controls treated with 5% DMSO. **Fig 8.** Analysis of viral and NS5 protein expressions from DENV WT and mutant replicons and virus. Immuno-fluorescence stainings for DENV dsRNA and NS5 protein were performed on BHK-21 cells at days 1–4 (D1-4) after electroporation of DENV-2 WT and mutant (A) replicon or (B) full length viral IVT RNA. Cells were fixed and the stated time-points and probed with mouse monoclonal anti-dsRNA (red; Scicons, USA) and rabbit polyclonal anti-NS5 (green; GeneTex, USA) primary antibodies, and goat anti-mouse-IgG- Alexa Fluor568 and goat anti-rabbit-IgG-FITC secondary antibodies (Invitrogen, USA). Nuclear DNA was stained with DAPI (blue; Thermofisher, USA). **Fig 9.** WNV (New York strain 3356) replicon cDNA was electroporated in BHK-21 cells, after which cells were seeded into 96-well plates and treated with compounds, **26i**, **27, 29** and **29i** (10-point, 3-fold serially diluted compounds from 0–50 μM), for 2 days. EC_50_ values from replicon cells were determined by measuring cellular renilla luciferase levels. All data points were measured in duplicates. **Table 1.** Data collection and refinement statistics of DENV3 FL NS5 co-crystals. **Table 2.** Analysis of DENV2 (strain NGC) WT and mutant replicons and virus replication.
(PDF)

## References

[1] Muso D, Gubler DJ (2016) Zika virus. Clin Microbiol Rev 29: 487–524. 10.1128/CMR.00072-15 27029595 PMC4861986

[2] Bhatt S, Gething PW, Brady OJ, Messina JP, Farlow AW, Moyes CL, et al (2013) The global distribution and burden of dengue. Nature 496: 504–507. 10.1038/nature12060 23563266 PMC3651993

[3] Capeding MR, Tran NH, Hadinegoro SR, Ismail HI, Chotpitayasunondh T, Chua MN, et al; CYD14 Study Group (2014) Clinical efficacy and safety of a novel tetravalent dengue vaccine in healthy children in Asia: a phase 3, randomised, observer-masked, placebo-controlled trial. Lancet 384: 1358–1365. 10.1016/S0140-6736(14)61060-6 25018116

[4] Sabchareon A, Wallace D, Sirivichayakul C, Limkittikul K, Chanthavanich P, Suvannadabba S, et al (2012) Protective efficacy of the recombinant, live-attenuated, CYD tetravalent dengue vaccine in Thai schoolchildren: a randomised, controlled phase 2b trial. Lancet 380: 1559–67. 10.1016/S0140-6736(12)61428-7 22975340

[5] Lim SP, Wang QY, Noble CG, Chen YL, Dong H, Zou B, et al (2013) Ten years of dengue drug discovery: progress and prospects. Antiviral Res 100: 500–519. 10.1016/j.antiviral.2013.09.013 24076358

[6] Mackenzie J (2005) Wrapping things up about virus RNA replication. Traffic 6: 967–977. 16190978 10.1111/j.1600-0854.2005.00339.x PMC7169867

[7] Miller S, Krijnse-Locker J (2008) Modification of intracellular membrane structures for virus replication. Nat Rev Microbiol 6: 363–374. 10.1038/nrmicro1890 18414501 PMC7096853

[8] Salonen A, Ahola T, Kaariainen L (2005) Viral RNA replication in association with cellular membranes. Curr Top Microbiol Immunol 285: 139–173. 15609503 10.1007/3-540-26764-6_5 PMC7120253

[9] Egloff MP, Benarroch D, Selisko B, Romette JL, Canard B (2002) An RNA cap (nucleoside-2'-O-)-methyltransferase in the flavivirus RNA polymerase NS5: crystal structure and functional characterization. EMBO J 21: 2757–2768. 12032088 10.1093/emboj/21.11.2757 PMC125380

[10] Ray D, Shah A, Tilgner M, Guo Y, Zhao Y, Dong H, et al (2006) West nile virus 5'-cap structure is formed by sequential guanine N-7 and ribose 2'-O methylations by nonstructural protein 5. J. Virol 80: 8362–8370. 16912287 10.1128/JVI.00814-06 PMC1563844

[11] Dong H, Ren S, Zhang B, Zhou Y, Puig-Basagoiti F, Li H, et al (2008) West Nile virus methyltransferase catalyzes two methylations of the viral RNA cap through a substrate-repositioning mechanism. J Virol 82: 4295–4307. 10.1128/JVI.02202-07 18305027 PMC2293060

[12] Chung KY, Dong H, Chao AT, Shi PY, Lescar J, Lim SP. (2010) Higher catalytic efficiency of N-7-methylation is responsible for processive N-7 and 2'-O methyltransferase activity in dengue virus. Virology 402: 52–60. 10.1016/j.virol.2010.03.011 20350738

[13] Daffis S, Szretter KJ, Schriewer J, Li J, Youn S, Errett J, et al (2010) 2'-O methylation of the viral mRNA cap evades host restriction by IFIT family members. Nature 468: 452–6. 21085181 10.1038/nature09489 PMC3058805

[14] Dong H, Chang DC, Hua MH, Lim SP, Chionh YH, Hia F, et al (2012) 2'-O methylation of internal adenosine by flavivirus NS5 methyltransferase. PLoS Pathog 8: e1002642 10.1371/journal.ppat.1002642 22496660 PMC3320599

[15] Zhao Y, Soh TS, Lim SP, Chung KY, Swaminathan K, Vasudevan SG, et al (2015) Molecular basis for specific viral RNA recognition and 2'-O-ribose methylation by the dengue virus nonstructural protein 5 (NS5). Proc Natl Acad Sci USA 112: 14834–14839. 10.1073/pnas.1514978112 26578813 PMC4672796

[16] Issur M, Geiss BJ, Bougie I, Picard-Jean F, Despins S, Mayette J, et al (2009) The flavivirus NS5 protein is a true RNA guanylyltransferase that catalyzes a two-step reaction to form the RNA cap structure. RNA 15: 2340–2350. 10.1261/rna.1609709 19850911 PMC2779676

[17] Bollati M, Milani M, Mastrangelo E, Ricagno S, Tedeschi G, Nonnis S, et al (2009) Recognition of RNA cap in the Wesselsbron virus NS5 methyltransferase domain: implications for RNA-capping mechanisms in Flavivirus. J Mol Biol 385(1): 140–152. 10.1016/j.jmb.2008.10.028 18976670

[18] Ackermann M, Padmanabhan R, (2001) De novo synthesis of RNA by the dengue virus RNA-dependent RNA polymerase exhibits temperature dependence at the initiation but not elongation phase. J. Biol. Chem. 276: 39926–39937. 11546770 10.1074/jbc.M104248200

[19] Nomaguchi M, Teramoto T, Yu L, Markoff L, Padmanabhan R, (2004) Requirements for West Nile virus (-)- and (+)-strand subgenomic RNA synthesis in vitro by the viral RNA dependent RNA polymerase expressed in Escherichia coli. J. Biol, Chem. 279: 12141–12151. 14699096 10.1074/jbc.M310839200

[20] Malet H, Egloff MP, Selisko B, Butcher RE, Wright PJ, Roberts M, et al (2007) Crystal structure of the RNA polymerase domain of the West Nile virus non-structural protein 5. J Biol Chem 282: 10678–10689. 17287213 10.1074/jbc.M607273200

[21] Yap TL, Xu T, Chen YL, Malet H, Egloff MP, Canard B, et al (2007) Crystal structure of the dengue virus RNA-dependent RNA polymerase catalytic domain at 1.85-angstrom resolution. J Virol 81: 4753–4765. 17301146 10.1128/JVI.02283-06 PMC1900186

[22] Noble CG, Lim SP, Chen YL, Liew CW, Yap L, Lescar J, et al (2013) Conformational flexibility of the Dengue virus RNA-dependent RNA polymerase revealed by a complex with an inhibitor. J Virol 87: 5291–5295. 10.1128/JVI.00045-13 23408636 PMC3624333

[23] Lim SP, Koh JH, Seh CC, Liew CW, Davidson AD, Chua LS, et al (2013) A crystal structure of the dengue virus non-structural protein 5 (NS5) polymerase delineates interdomain amino acid residues that enhance its thermo-stability and de novo initiation activities. J Biol Chem 288: 31105–31114. 10.1074/jbc.M113.508606 24025331 PMC3829423

[24] Potisopon S, Priet S, Collet A, Decroly E, Canard B, Selisko B, et al (2014) The methyltransferase domain of dengue virus protein NS5 ensures efficient RNA synthesis initiation and elongation by the polymerase domain. Nucleic Acids Res 42: 11642–11656. 10.1093/nar/gku666 25209234 PMC4191377

[25] Zhao Y, Soh S, Zheng J, Chan KWK, Phoo WW, Lee CC, et al (2015) A crystal structure of the dengue virus NS5 protein reveals a novel inter-domain interface essential for protein flexibility and virus replication. PLOS Pathogens 11(3):e1004682 10.1371/journal.ppat.1004682 25775415 PMC4361662

[26] Ashour J, Laurent-Rolle M, Shi PY, Garcia-Sastre A (2009) NS5 of dengue virus mediates STAT2 binding and degradation. J Virol 83: 5408–5418. 10.1128/JVI.02188-08 19279106 PMC2681973

[27] Hannemann H, Sung PY, Chiu HC, Yousuf A, Bird J, Lim SP, et al (2013) Serotype-specific differences in dengue virus non-structural protein 5 nuclear localization. J Biol Chem 288: 22621–22635. 10.1074/jbc.M113.481382 23770669 PMC3829348

[28] Sousa R (1996) Structural and mechanistic relationships between nucleic acid polymerases. Trends Biochem Sci 21: 186–190. 8871404

[29] Caillet-Saguy C, Lim SP, Shi P-Y, Lescar J, Bressanelli S (2014) Polymerases of hepatitis C viruses and flaviviruses: Structural and mechanistic insights and drug development. Antiviral Res 105:8–16. 10.1016/j.antiviral.2014.02.006 24561230

[30] Keller TH, Chen YL, Knox JE, Lim SP, Ma NL, Patel SJ, et al (2006) Finding new medicines for flaviviral targets. Novartis Found Symp 277: 102–114; discussion 114–109; 251–103. 17319157

[31] Noble CG, Shi PY (2012) Structural biology of dengue virus enzymes: towards rational design of therapeutics. Antiviral Res 96: 115–126. 10.1016/j.antiviral.2012.09.007 22995600

[32] Lim SP, Noble CG, Shi PY (2015) The dengue virus NS5 protein as a target for drug discovery. Anti-viral Res 119: 57–67. 10.1016/j.antiviral.2015.04.010 25912817

[33] Yin Z, Chen YL, Schul W, Wang QY, Gu F, Duraiswamy J, et al (2009a) An adenosine nucleoside inhibitor of dengue virus. Proc Natl Acad Sci USA 106: 20435–20439. 19918064 10.1073/pnas.0907010106 PMC2787148

[34] Yin Z, Chen YL, Kondreddi RR, Chan WL, Wang G, Ng RH, et al (2009b) N-sulfonylanthranilic acid derivatives as allosteric inhibitors of dengue viral RNA-dependent RNA polymerase. J Med Chem 52: 7934–7937. 20014868 10.1021/jm901044z

[35] Niyomrattanakit P, Chen YL, Dong H, Yin Z, Qing M, Glickman JF, et al (2010). Inhibition of dengue virus polymerase by blocking of the RNA tunnel. J Virol 84: 5678–5686. 10.1128/JVI.02451-09 20237086 PMC2876596

[36] Smith TM, Lim SP, Yue K, Busby SA, Arora R, Seh CC, et al (2014) Identifying Initiation and Elongation Inhibitors of Dengue Virus RNA Polymerase in a High-Throughput Lead-Finding Campaign. J Biomol Screen 20: 153–163. 10.1177/1087057114551141 25252731

[37] Noble CG, Lim SP, Arora R, Yokokawa F, Nilar N, Seh CC, et al (2016) A conserved pocket in the dengue virus polymerase identified through fragment-based screening. J. Biol Chem 291: 8541–8548. 10.1074/jbc.M115.710731 26872970 PMC4861426

[38] Yokokawa F, Shahul N, Noble CG, Lim SP, Rao R, Tania S, et al (2016) Discovery of Potent Non-Nucleoside Inhibitors of Dengue Viral RNA-Dependent RNA Polymerase From a Fragment Hit Using Structure-Based Drug Design. J. Med Chem 59: 3935–3952 10.1021/acs.jmedchem.6b00143 26984786

[39] Niyomrattanakit P, Wan KF, Chung KY, Abas SN, Seh CC, Dong H, et al (2015) Stabilization of dengue virus polymerase in de novo initiation assay provides advantages for compound screening. Antiviral Res 119: 36–46. 10.1016/j.antiviral.2015.04.007 25896272

[40] Ng C, Gu F, Phong WY, Chen Y, Lim SP, Davidson A, et al (2007) Construction and Characterization of a Stable Subgenomic Dengue virus type 2 Replicon System for antiviral compound and siRNA screening. Antiviral Res 76: 222–231. 17662475 10.1016/j.antiviral.2007.06.007

[41] Molina MD, Jafari R, Ignatushchenko M, Seki T, Larsson EA, Dan C, Sreekumar L, Cao Y, Nordlund P (2013) Monitoring drug target engagement in cells and tissues using the cellular thermal shift assay. Science 341: 84–87. 10.1126/science.1233606 23828940

[42] Sievers F, Wilm A, Dineen DG, Gibson TJ, Karplus K, Li W, et al (2011). Fast, scalable generation of high-quality protein multiple sequence alignments using Clustal Omega. Molecular Systems Biology 7: 539 10.1038/msb.2011.75 21988835 PMC3261699

[43] Blight KJ, Kolykhalov AA, Rice CM (2000) Efficient initiation of HCV RNA replication in cell culture. Science 290: 1972–1974. 11110665 10.1126/science.290.5498.1972

[44] Sofia MJ, Chang W, Furman PA, Mosley RT, Ross BS (2012) Nucleoside, nucleotide, and non-nucleoside inhibitors of hepatitis C virus NS5B RNA-dependent RNA-polymerase. J Med Chem 55: 2481–2531. 10.1021/jm201384j 22185586

[45] Malet H, Massé N, Selisko B, Romette JL, Alvarez K, Guillemot JC et al (2008) The flavivirus polymerase as a target for drug discovery. Antiviral Res. 80: 23–35. 10.1016/j.antiviral.2008.06.007 18611413

[46] Surana P, Satchidanandam V, Nair DT (2014) RNA-dependent RNA polymerase of Japanese encephalitis virus binds the initiator nucleotide GTP to form a mechanistically important pre-initiation state. Nucleic Acids Res 42: 2758–2773. 10.1093/nar/gkt1106 24293643 PMC3936712

[47] Klema VJ, Ye M, Hindupur A, Teramoto T, Gottipati K, Padmanabhan R, et al (2016) PLoS Pathog. 12(2):e1005451 10.1371/journal.ppat.1005451 26895240 PMC4760774

[48] Selisko B, Potisopon S, Agred R, Priet S, Varlet I, Thillier Y, et al (2012) Molecular basis for nucleotide conservation at the ends of the dengue virus genome. PLoS Pathog 8: e1002912 10.1371/journal.ppat.1002912 23028313 PMC3441707

[49] Winn MD, Ballard CC, Cowtan KD, Dodson EJ, Emsley P, Evans PR, et al (2011) Overview of the CCP4 suite and current developments. Acta Crystallogr D Biol Crystallogr 67: 235–242. 10.1107/S0907444910045749 21460441 PMC3069738

[50] Wang QY, Dong H, Zou B, Karuna R, Wan KF, Zou J, et al (2015) Discovery of Dengue Virus NS4B Inhibitors. J Virol 89: 8233–8244. 26018165 10.1128/JVI.00855-15 PMC4524232

