# Supplementary material for: Potent Allosteric Dengue Virus NS5 Polymerase Inhibitors: Mechanism of Action and Resistance Profiling
Source: PLoS Pathog. 2016 Aug 8;12(8):e1005737. doi: 10.1371/journal.ppat.1005737 (PMC4976923; doi:10.1371/journal.ppat.1005737)
Supplement: S1 Text — Fig 1A. Representative IC50 curves for N-pocket inhibitors tested in DENV4 FL NS5 de novo initiation FAPA assay [3]. Briefly, compounds (10-point, 3-fold serially diluted compounds from 0–20 or 100 μM) were incubated at RT for 20 min with enzyme alone, in 384-well plate opaque plates, after which reactions were started with addition of ssRNA and nucleotide substrate components, and allowed to proceed for 2 hr. Reactions were stopped by addition of 10 μl of 2.5X STOP buffer with 25 nM CIP, re- incubated at RT for 60 min and read on a Tecan Safire II microplate reader (excitationmax and emissionmax wavelengths 422 nm and 566 nm). Data was fitted to the four parameter logistic equation and IC50 curves plotted using Graphpad® Prism software. Table contains average IC50 values and hill slopes obtained. All data points were measured in duplicates. Fig 1B. Representative EC50 and CC50 curves for N-pocket inhibitors tested in HuH-7 DENV-2 replicon cell-based assay [1]. Cells were seeded over-night in white opaque 384-well plates followed by incubation with increasing compound concentrations (10-point, 2-fold serially diluted compounds from 0–50 μM) for 48 hr, after which cellular renilla luciferase (EC50) or ATP (CC50) levels, measured as relative light units (RLU), were determined using ViviRen and Cell Titer-Glo (Promega) according to manufacturer’s protocol. All data points were measured in duplicates. Fig 2. Enzyme inhibition kinetics of N-pocket compounds against DENV polymerase. DENV4 FL NS5 dnI FAPA assays [5] were performed in increasing concentrations of N-pocket inhibitor, 15 or 29 or 3’dGTP (control) with 0–500 nM RNA or 0–50 μM GTP to determine mechanism of inhibition with respect to either suibstrates. Representative Michaelis-Menton plots were derived from non-linear regression curve fitting using Graphpad Prism software. Fig 3. Crystal structures of DENV3 FL NS5 were obtained by co-crystallization according to conditions from reference 7 as described in Suppl [file ppat.1005737.s001.pdf]

SUPPORTING INFORMATION

S1 TEXT

Potent allosteric dengue virus NS5 polymerase inhibitors: mechanism of action and resistance profiling

Siew Pheng Lim[1,*], Christian Noble[1], Cheah Chen Seh[1], Tingjin Sherryl Soh[1,2], Abbas El Sahili[2], Kar Yarn Grace Chan[2], Julien Lescar[2,3], Rishi Arora[4], Timothy Benson[4], Shahul Nilar[1], Ujjini Manjunatha[1], Kah Fei Wan[1], Hongping Dong[1], Xie Xuping[1,#], Pei-Yong Shi[1,#], Fumiaki Yokokawa[1]

[1]Novartis Institute for Tropical Diseases, #05-01 Chromos, 10 Biopolis Road, Singapore 138670

[2]School of Biological Sciences, Nanyang Technological University. 60 Nanyang Drive, Singapore 637551

[3]UPMC UMRS CR7 - CNRS ERL 8255-INSERM U1135 Centre d'Immunologie et des Maladies Infectieuses. Centre Hospitalier Universitaire Pitié-Salpêtrière, Faculté de Médecine Pierre et Marie Curie, Paris, France

[4]Novartis Institute for Biomedical Research, 250 Massachusetts Avenue, Cambridge, MA 02139, USA

[#]Present address: Department of Biochemistry & Molecular Biology, Sealy Center for Structural Biology & Molecular Biophysics, University of Texas Medical Branch, Galveston, TX 77555, USA.

*To whom correspondence may be addressed: siew_pheng.lim@novartis.com; Tel: 65-67222924.

Supporting Methods.

Compound testing with DENV *dn*I FAPA assays. Final *dn*I assay reaction mixtures contained 100 nM DENV 5´UTR-L-3´UTR IVT RNA, 20 µM ATP, 20 µM GTP, 20 µM UTP, 5 µM ATTO-CTP, and 100 nM of DENV FL NS5 protein in 15 µl in assay buffer comprising 50 mM Tris/HCl, pH 7.5, 10 mM KCl, 1 mM $MgCl_2$, 0.3 mM $MnCl_2$ (DENV2 and 4) or 1 mM $MnCl_2$ (DENV1 and 3), 0.001% Triton-X-100 and 10 µM cysteine [1]. To perform dose-response inhibition measurements, compounds from 0-20 or -100 µM concentrations are two-fold serially diluted into 384-well black opaque plates (Corning Costar), after which 100 nM DENV FL NS5 protein was added into respective wells. The plates were sealed, briefly shaken and centrifuged at 1500 rpm for 30 sec and incubated at RT for 15 min. Thereafter, RNA and ATTO-CTP, ATP, GTP and UTP were added to the wells to start the reactions. The plates were re-sealed, shaken and centrifuged as before, followed by incubation RT for 120 min. Reactions were stopped by addition of 10 µl of 2.5X STOP buffer (200 mM NaCl, 25 mM $MgCl_2$, 1.5M DEA, pH 10; Promega) with 25 nM calf intestinal phosphatase (CIP), re- incubation at RT for 60 min and read on a Tecan Safire II microplate reader. For order-of-addition experiments, DENV4 FL NS5 was incubated for one hour at RT with RNA, ATP, and GTP or RNA, ATP, GTP and ATTO-CTP, followed by exposure to serially diluted compounds for 20 min at RT. The missing components (ATTO-CTP and UTP or UTP alone) were added and the reactions continued for 120 min after which STOP buffer was added as before. All datapoints were performed in duplicate wells. Each compound was tested at least twice.

Compound testing in DENV1-4 infected cells by high content imaging. Approximately 7 $\times 10^3$ A549 cells per well were seeded into 384-well plates in Ham's F-12K medium

containing 2 % FBS, 1mM L-glutamine and 1 % penicillin-streptomycin and incubated over-night at 37 °C in 5 % CO_2. At 24 h post-seeding, cells were infected with DENV1-4 at multiplicity of infection [MOI] of 0.3-1 (DENV1, strain MY97-10245, MOI = 0.5; DENV2, strain MY97-10340, MOI = 0.3; DENV3 strain MY05-34640, MOI = 1; DENV4, strain MY01-22713; MOI = 0.5) and treated immediately with 10-point, 3-fold serially diluted compounds (at final 0.5% DMSO concentration; 4). At 48 h post-infection, cells were fixed with 4 % paraformaldehye and stained with 4G2 antibody conjugated with Dylight 488 (for DENV envelope protein) and DRAQ5 (for nuclear DNA). Quantifications of DENV envelope protein were determined using the Opera high content imaging system and analysis software (Perkin Elmer, USA). Calculation of EC_{50} values were performed using Helios software package (Novartis, Basel).

Compound testing in DENV2 and HCV sub-genomic replicon assays. A549, BHK-21 and Huh7 DENV2 sub-genomic replicon cells [2] were seeded at a density of 3,000 cells per well in a 384-well microplate. After incubation at 37 °C with 5 % CO_2 overnight, the cells were treated with serially diluted compounds in a 10-point dose response starting from 20 or 50 μM concentrations. At 48 hr of post-incubation, renilla luciferase activities were measured with the EndurRen live-cell substrate (Promega, USA) according to the manufacturer's protocol. Following luciferase activity measurements, the CellTiter-Glo reagent (Promega, USA) was added to determine the cytotoxic effects of the compounds. For the HCV replicon assay, Huh-7.5 cells harboring the HCV replicon [3] were seeded at a density of 20,000 cells per well in a 96-well microplate. At 48 hr after compound treatment, the cells were assayed for firefly luciferase activity by using a Bright-Glo luciferase assay (Promega, USA). NITD-008, a nucleoside inhibitor of DENV and HCV [4], was included as a control. Compounds

were tested up to 25 μM in HCV replicon cells due to limits in DMSO tolerability of these cells, and up to 50 μM in other cell types, based on compound solubility.

Compound testing in cells transiently expressing DENV and WNV replicon. Renilla luciferase sub-genomic replicon of DENV2 (strain New Guinea C) or WNV (New York strain 3356) cloned into the pACYC plasmid, was *in vitro* transcribed using a T7 mMessage mMachine kit (Ambion, Austin, TX) after linearization with ClaI, as described previously [5]. A549 cells were electroporated with 10 μg of replicon RNA using a GenePulser Xcell system (Bio-Rad, Hercules, CA) with 3 pulses, at 450 V, 25 μF, at 5-10 sec intervals. Transfected cells were seeded into a 96-well plate at 1.5×10^4 cells per well, followed by treatment with the compound or the dimethyl sulfoxide (DMSO) control and incubation at 37°C in 5 % CO_2. At various time points post-transfection, cells were washed once with phosphate-buffered saline (PBS) and lysed in 20 μl 1× lysis buffer (Promega, USA) and assayed for luciferase signals with a Clarity luminescence microplate reader (BioTek, USA) using the renilla luciferase assay system (Promega, USA). CellTiter-Glo reagent was added to the lysates to determine the cytotoxicity effects of compounds. Duplicate wells were seeded for each data point.

Compound testing in cells transiently expressing infectious DENV. Full length infectious virus of DENV2 (strain New Guinea C) cloned into the pACYC plasmid was *in vitro* transcribed using a T7 mMessage mMachine kit (Ambion, Austin, TX) after linearization with ClaI, as described previously [5]. BHK-21 cells were electroporated with 10 μg of replicon RNA using a GenePulser Xcell system (Bio-Rad, Hercules, CA) with 3 pulses, at 850 V, 25 μF, at 5-10 sec intervals. Transfected cells were seeded into 96-well plates (1×10^5 cells per well), followed by treatment with the compound or the dimethyl sulfoxide

(DMSO) control and incubated at 37 °C in 5 % CO_2. At various time points post-transfection (p.t.), cells were washed once with phosphate-buffered saline (PBS) and lysed in 20 µl 1× lysis buffer (Promega, USA) and assayed for luciferase signals with a Clarity luminescence microplate reader (BioTek, USA). Cell viability was measured by using CellTiter-Glo kit (Promega, USA) according to the manufacturer's protocols. Absorbance was measured at 450 nm by using a microplate reader (Tecan). Duplicate wells were seeded for each data point.

Plaque assay. Virus stock was produced by harvesting the supernatant of DENV2 full-length RNA-transfected BHK-21 cells at 24, 48, 72, 96, and 120 hours post transfection. Virus titer and morphology were determined by standard plaque assay. Briefly, a series of 10-fold dilutions was prepared by first diluting 50 µl virus stock with 450 µl RPMI 1640 media containing 2 % FBS to obtain 10^{-1} dilution and then further diluted until a final 10^{-6} dilution was achieved. Confluent BHK-21 cells (1×10^5 cells per well, plated 2 days in advance) grown in 24-well plate was added with 200 µl of each dilution per well. Duplicates were prepared for each time point and dilution factor. The infection was allowed to proceed at 30°C for 1 hour, followed by removing the media and adding 500 µl 0.8% methyl-cellulose overlay (containing RPMI, 2 % FBS, 1 % P/S, 0.05 % $NaHCO_3$, 25 mM Na Hepes, and 0.5 % DMSO) into each well. The plates were incubated for 5 days at 37 °C in 5 % CO_2 before fixing in 3.7 % formaldehyde and staining with 1 % crystal violet. The viral titer was calculated as plaque-forming unit (PFU) per ml.

Immunofluorescence assay (IFA). Cells harbouring DENV replicons or infectious virus were seeded into an eight-well Lab-Tek chamber slide (Thermo Fisher Scientific) and incubated at 37 °C in 5 % CO_2. At various time-points, cells were washed twice in PBS, and then fixed in cold methanol at -20 °C. Cells were blocked with PBS containing 1 % bovine

serum albumin, 1 % FBS and 0.05 % Tween-20, and washed thrice with PBS followed by incubation with anti-rabbit DENV NS5 (GeneTex) or anti-mouse dsRNA (English & Scientific Consulting Kft., Hungary), After 1 hr, cells were washed thrice with PBS, and added with secondary antibodies, Alexa Fluor 488 goat anti-rabbit IgG or Alexa Fluor 568 donkey anti-mouse IgG (Invitrogen, USA). After washes with PBS, the cells were mounted in mounting medium with 4′,6-diamidino-2-phenylindole (DAPI; Vector Laboratories, Inc.). Fluorescence images were acquired with a Leica DM4000 B system.

Data visualization and analysis. Assay development data were generated in Microsoft Excel. Data analysis and visualization was done with GraphPad Prism5 and Spotfire. Positive (maximum RFU) and negative (minimum RFU) controls were averaged and subtracted from sample data to calculate % inhibition by using equation 1 below:

% inhibition = (100 – ((sample signal – average Negative)/(average Positive – average Negative)×100))

The four parameter logistic equation was used for curve fitting to calculate IC_{50} according to equation 2 below:

$$Y = Bottom + (Top - Bottom)/(1+((10^\wedge(logIC_{50} - X)^\wedge Hill\ slope))$$

where Bottom is the minimum Y value, Top is the maximum Y value, and Hill slope is the slope of the linear portion of the semi-log curve. IC_{50} was extrapolated from $logIC_{50}$ according to the GraphPad algorithm.

Co-crystallization of compounds with DENV3 FL NS5. Co-crystallization of either compounds **27** or **29** with DENV3 FL NS5 was performed by hanging drop method mixing 1µl of mother liquor (16-18 % PEG 3350, 0.2 M Mg Acetate, 1 mM TCEP) to 1 µL of protein mixture (2-3 mg/ml of protein with final concentration of 1-5 mM of each compound diluted in 10 % DMSO).

Supporting Figures.

Fig. 1A. Representative IC_{50} curves for N-pocket inhibitors tested in DENV4 FL NS5 de novo initiation FAPA assay [1]. Briefly, compounds (10-point, 3-fold serially diluted compounds from 0- 20 or 100 μM) were incubated at RT for 20 min with enzyme alone, in 384-well plate opaque plates, after which reactions were started with addition of ssRNA and nucleotide substrate components, and allowed to proceed for 2 hr. Reactions were stopped by addition of 10 µl of 2.5X STOP buffer with 25 nM CIP, re- incubated at RT for 60 min and read on a Tecan Safire II microplate reader ($excitation_{max}$ and $emission_{max}$ wavelengths 422 nm and 566 nm). Data was fitted to the four parameter logistic equation and IC_{50} curves plotted using Graphpad® Prism software. Table contains average IC_{50} values and hill slopes obtained. All data points were measured in duplicates.

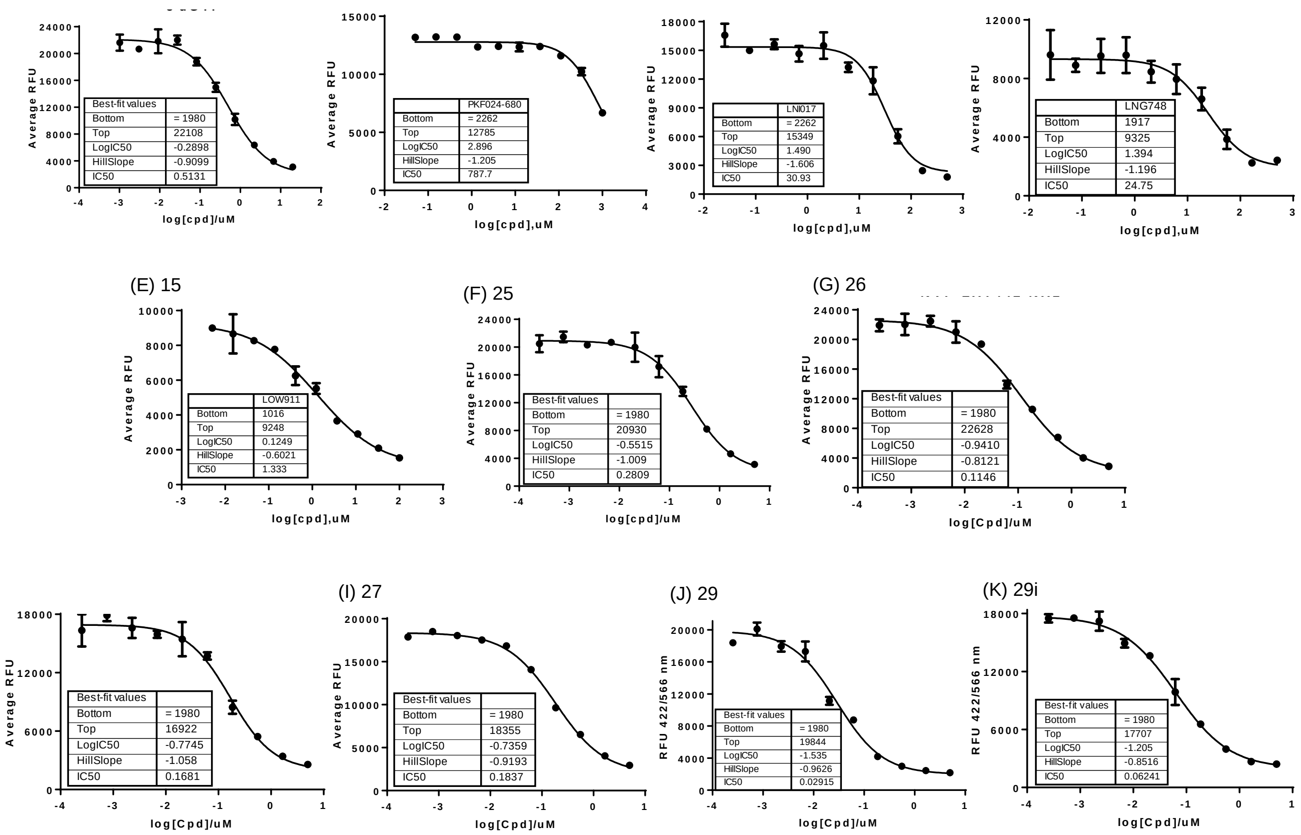

Fig. 1B. Representative EC_{50} and CC_{50} curves for N-pocket inhibitors tested in HuH-7 DENV-2 replicon cell-based assay [2]. Cells were seeded over-night in white opaque 384-well plates followed by incubation with increasing compound concentrations (10-point, 2-fold serially diluted compounds from 0-50 μM) for 48 hr, after which cellular renilla luciferase (EC_{50}) or ATP (CC_{50}) levels, measured as relative light units (RLU), were determined using ViviRen and Cell Titer-Glo (Promega) according to manufacturer's protocol. All data points were measured in duplicates.

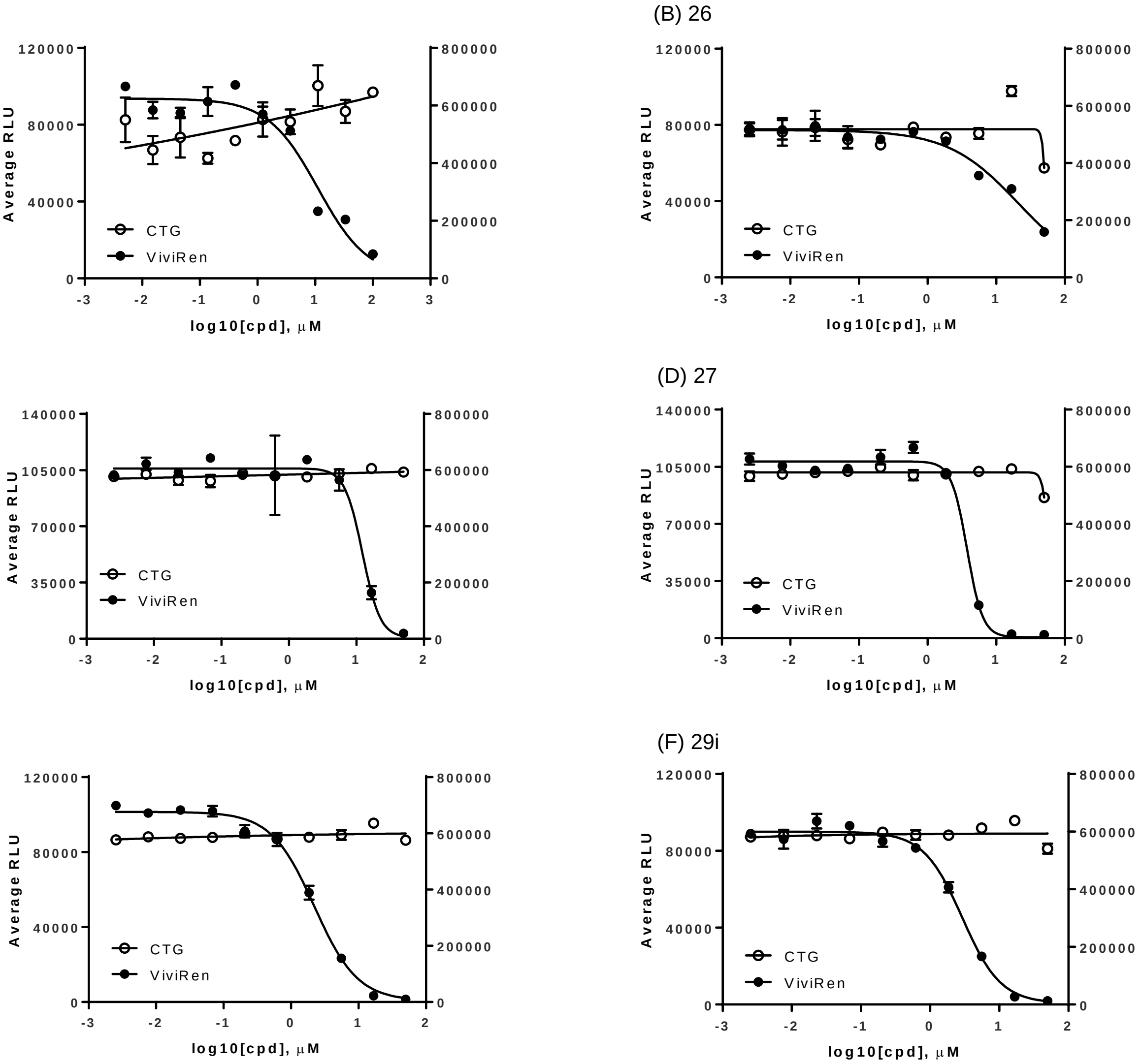

Fig. 2. Enzyme inhibition kinetics of N-pocket compounds against DENV polymerase. DENV4 FL NS5 *dn*I FAPA assays [1] were performed in increasing concentrations of N-pocket inhibitor, **15** or **29** or 3’dGTP (control) with 0-500 nM RNA or 0-50 μM GTP to determine mechanism of inhibition with respect to either suibstrates. Representative Michaelis-Menton plots were derived from non-linear regression curve fitting using Graphpad Prism software.

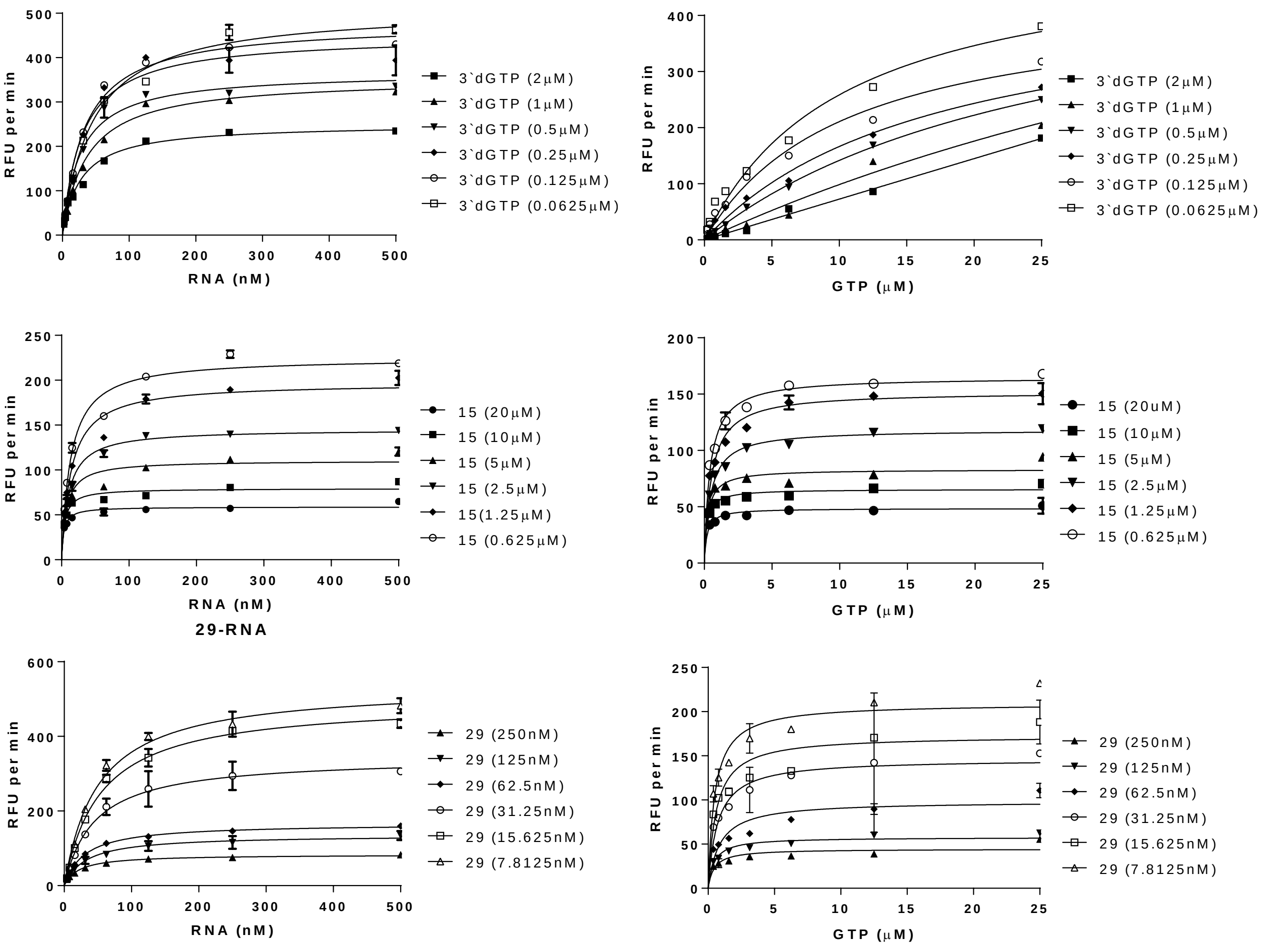

Fig. 3. Crystal structures of DENV3 FL NS5 were obtained by co-crystallization according to conditions from [6] as described in Supplementary Materials. (A) Overall view of the DENV3 FL NS5 structure displayed as ribbon with the methyltransferase domain in red, the linker region in orange, palm, thumb and fingers subdomains colored in olive, green and blue respectively. Both compounds **23** (magenta sticks) and **29** (yellow sticks) are overlaid in the polymerase domain. Magnified views of compound **29** (B) and **27** (C) with Fo-Fc contoured at 4 σ where each compound was omitted from the phase calculation. (D) Superimposition of the RdRp domain from FL NS5 (green ribbon) and RdRp (pink ribbon) with bound compounds **29** and **23** in sticks showing the absence of conformational changes. (E) Superimposition of compound **29** bound to FL NS5 structure (magenta sticks) and bound to RdRp structure (pink sticks) and compound **23** (panel F) bound to FL NS5 (grey sticks) and bound to RdRp (orange sticks). The compound conformations are closely superimposable. PDB codes for the FL NS5 structures with compounds 27 and 29 are 5JJS and 5JJR, respectively.

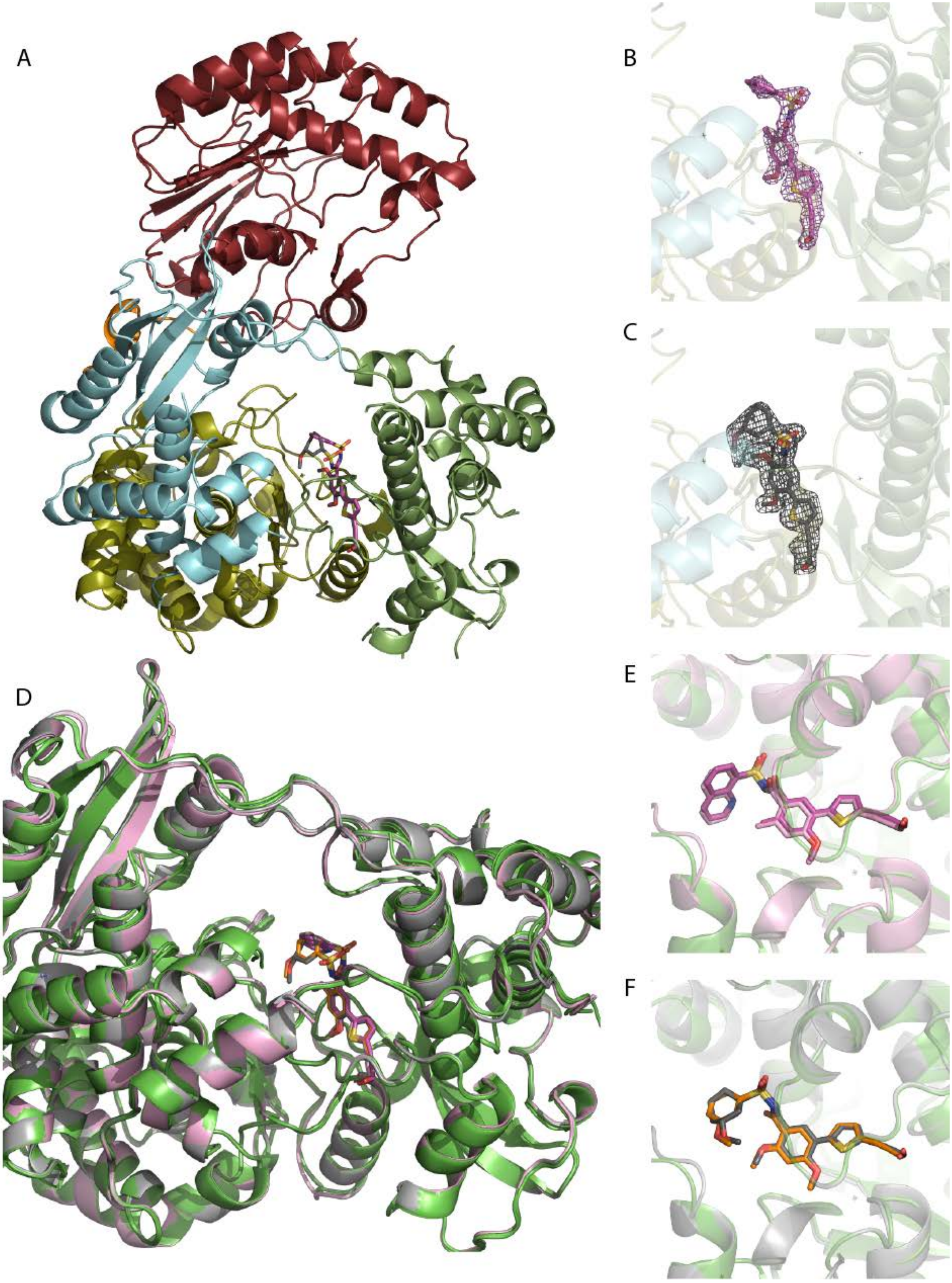
A
B
C
D
E
F

Fig. 4. Phylogenetic tree representing relatedness of N-pocket amino acid residues from different members of the Flavivirus family, derived from Clustal Omega program [8].

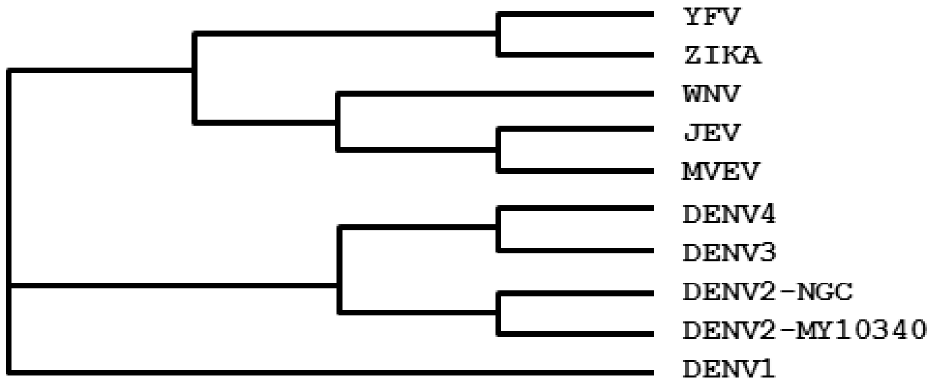

Fig. 5. Effects of N-pocket compounds on DENV polymerase thermo-stability. Melting temperature (T_m) was assessed by thermo-denaturation in presence of the SYPRO Orange dye as described in Materials and Methods. (A-F) Representative melting curves of DENV4 RdRp domain (aa 266-900; A-C) and FL NS5 (D-F) in presence of 50 μM N-pocket inhibitors or 5 % DMSO control. (G) Cellular thermal shifts assays [9] were performed with BHK-21 DENV2-NGC replicon cell lysates and 40 mM of **27** or **29**. Briefly, lysates were incubated with compounds or 5 % DMSO for 1 hr at 4 ^{o}C, followed by heating at 30-70 ^{o}C. Samples were spun and the supernatants loaded onto 12 % SDS-PAGE gels, followed by gel electrophoresis and western blotting with anti-DENV2 NS5 antibody (GeneTex, USA). Table shows the changes in protein melting temperatures in presence of compounds compared to controls treated with DMSO.

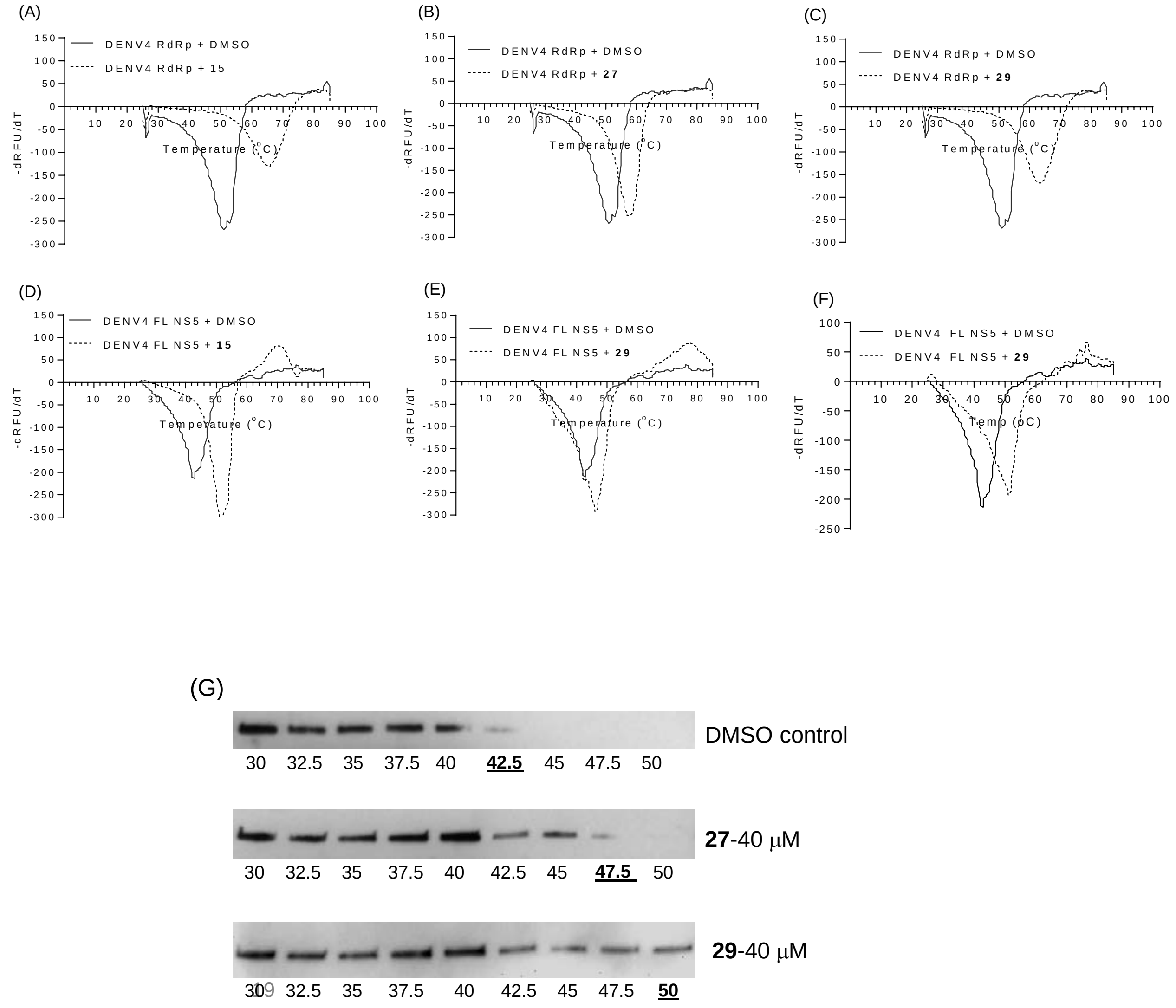

(A)
DENV4 RdRp + DMSO
DENV4 RdRp + 15
-dRFU/dT
Temperature (°C)
(B)
DENV4 RdRp + DMSO
DENV4 RdRp + 27
-dRFU/dT
Temperature (°C)
(C)
DENV4 RdRp + DMSO
DENV4 RdRp + 29
-dRFU/dT
Temperature (°C)
(D)
DENV4 FL NS5 + DMSO
DENV4 FL NS5 + 15
-dRFU/dT
Temperature (°C)
(E)
DENV4 FL NS5 + DMSO
DENV4 FL NS5 + 29
-dRFU/dT
Temperature (°C)
(F)
DENV4 FL NS5 + DMSO
DENV4 FL NS5 + 29
-dRFU/dT
Temp (oC)
(G)
DMSO control
30 32.5 35 37.5 40 42.5 45 47.5 50
27-40 μM
30 32.5 35 37.5 40 42.5 45 47.5 50
29-40 μM
30 32.5 35 37.5 40 42.5 45 47.5 50

Fig. 6. Activity profiles of DENV polymerase bearing resistant phenotype amino acid changes. Recombinant DENV2 (A, C) and DENV4 (B, D) FL NS5 proteins bearing single or double amino acid changes in the N-pocket were tested in *de novo* initiation (A, B) and elongation (C, D) FAPA assays and compared against activities of WT DENV2 or DENV4 FL NS5 proteins. Reactions were conducted over 2 hr at RT and from average relative fluorescence units (RFU) obtained from one experiment. All data points were measured in triplicate.

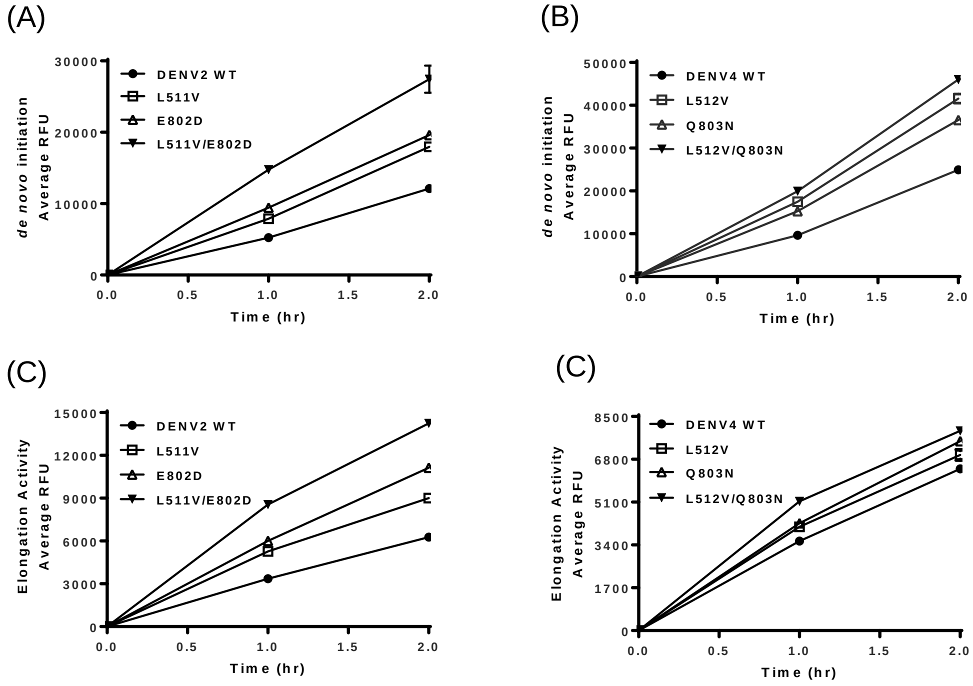

Fig. 7. Effects of compounds on DENV polymerase thermo-stability. Melting temperature (T_m) was assessed by thermo-denaturation in presence of the SYPRO Orange dye as described in Materials and Methods. (A-D) Representative melting curves of *in vitro* expressed recombinant DENV2 FL NS5 WT or mutant proteins in presence of 50 μM compound or 5 % DMSO control. Table shows the melting temperatures of DENV-2 and -4 FL NS5 WT and mutant proteins as well as the changes in their melting temperatures in presence of compounds compared to controls treated with 5 % DMSO.

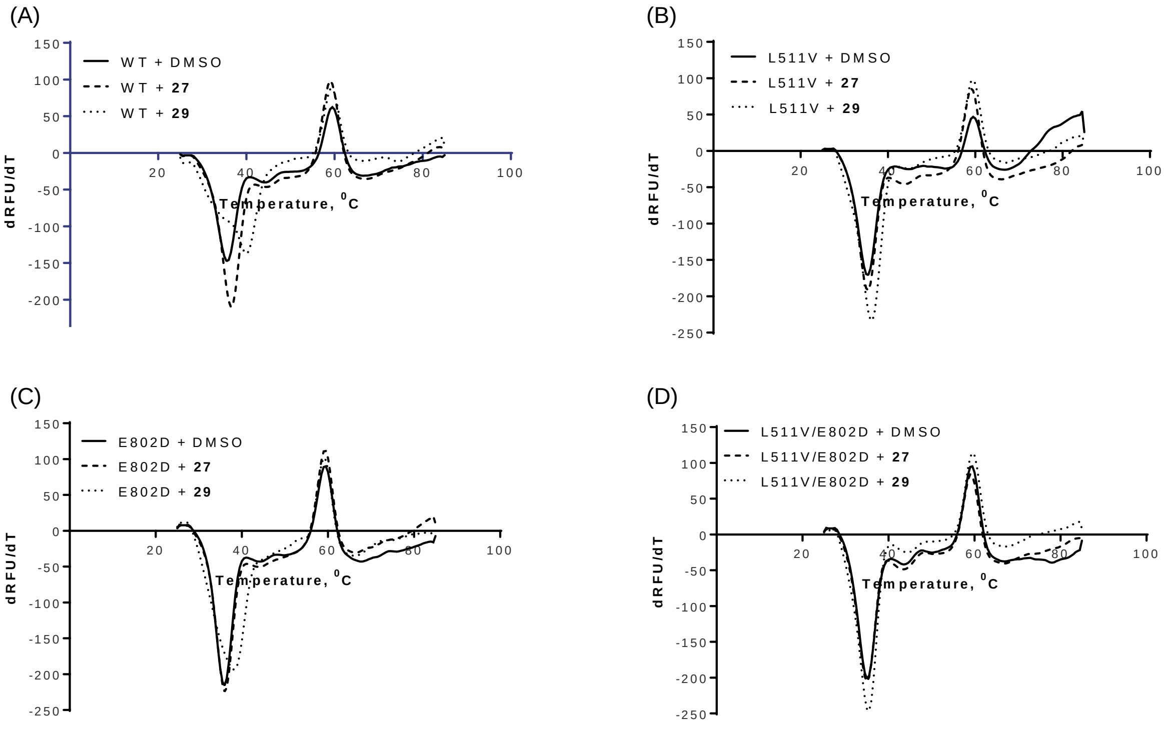

	Protein melting temperatures ,T_m (°C) [T_m change compared to DMSO control]							
	DENV2 FL NS5				DENV4 FL NS5			
	WT	L511V	E802D	L511V/E802D	WT	L512V	Q802N	L512V/Q802N
+ DMSO	35.5	35.5	36	35	37.5	36.5	37	36
+ **27**	37 [1.5]	35.5 [0]	36 [0]	35 [0]	38.5 [1]	36.5 [0]	37.5 [0.5]	35 [-1]
+ **29**	40 [4.5]	36.5 [1.0]	38 [2.0]	35.5 [0.5]	40.5 [3]	38 [1.5]	38.5 [1.5]	35.5 [-0.5]

Fig. 8. Analysis of viral and NS5 protein expressions from DENV WT and mutant replicons and virus. Immuno-fluorescence stainings for DENV dsRNA and NS5 protein were performed on BHK-21 cells at days 1-4 (D1-4) after electroporation of DENV-2 WT and mutant (A) replicon or (B) full length viral IVT RNA. Cells were fixed and the stated time-points and probed with mouse monoclonal anti-dsRNA (red; Scicons, USA) and rabbit polyclonal anti-NS5 (green; GeneTex, USA) primary antibodies, and goat anti-mouse-IgG-Alexa Fluor568 and goat anti-rabbit-IgG-FITC secondary antibodies (Invitrogen, USA). Nuclear DNA was stained with DAPI (blue; Thermofisher, USA).

(A)

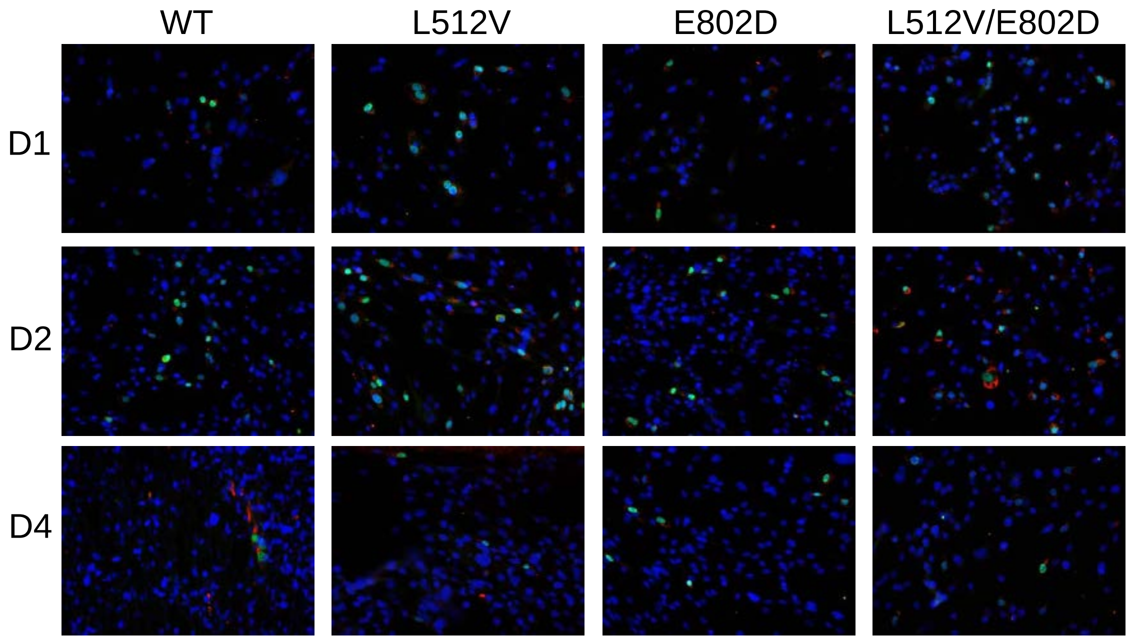

(B)

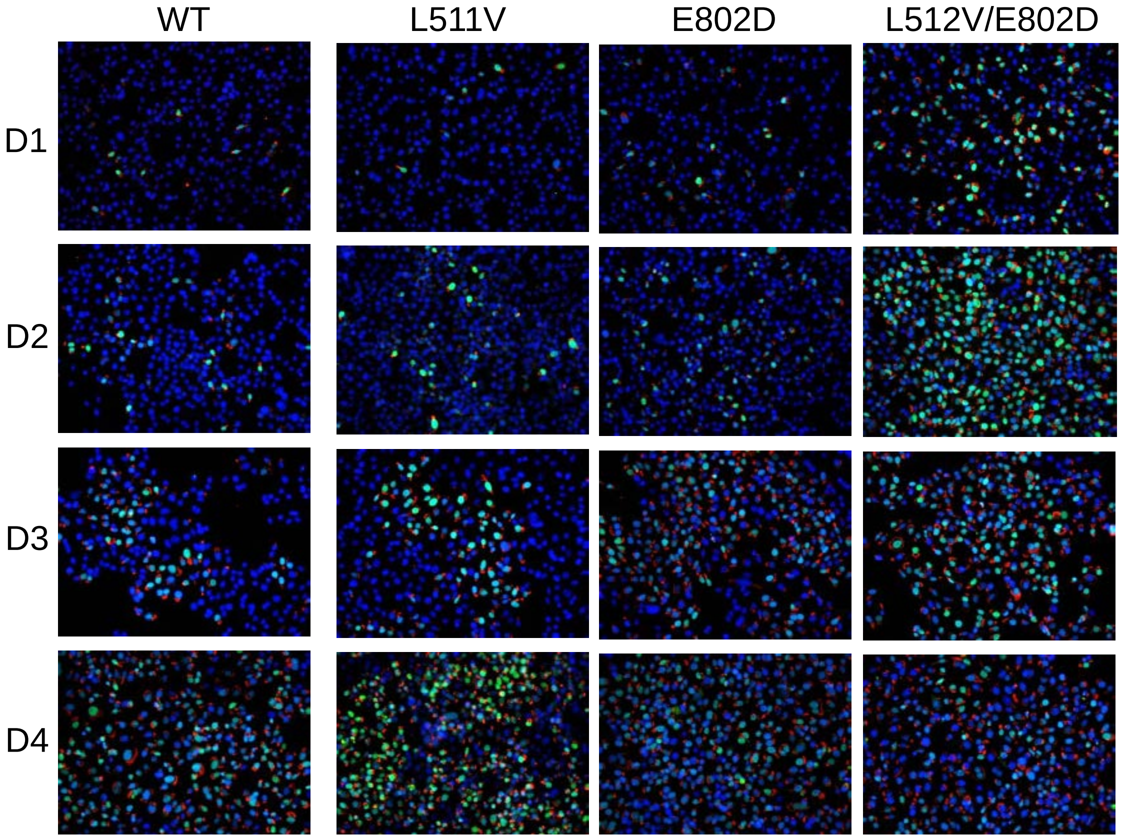

Fig. 9. WNV (New York strain 3356) replicon cDNA was electroporated in BHK-21 cells, after which cells were seeded into 96-well plates and treated with compounds, **26i**, **27, 29** and **29i** (10-point, 3-fold serially diluted compounds from 0- 50 μM), for 2 days. EC_{50} values from replicon cells were determined by measuring cellular renilla luciferase levels. All data points were measured in duplicates.

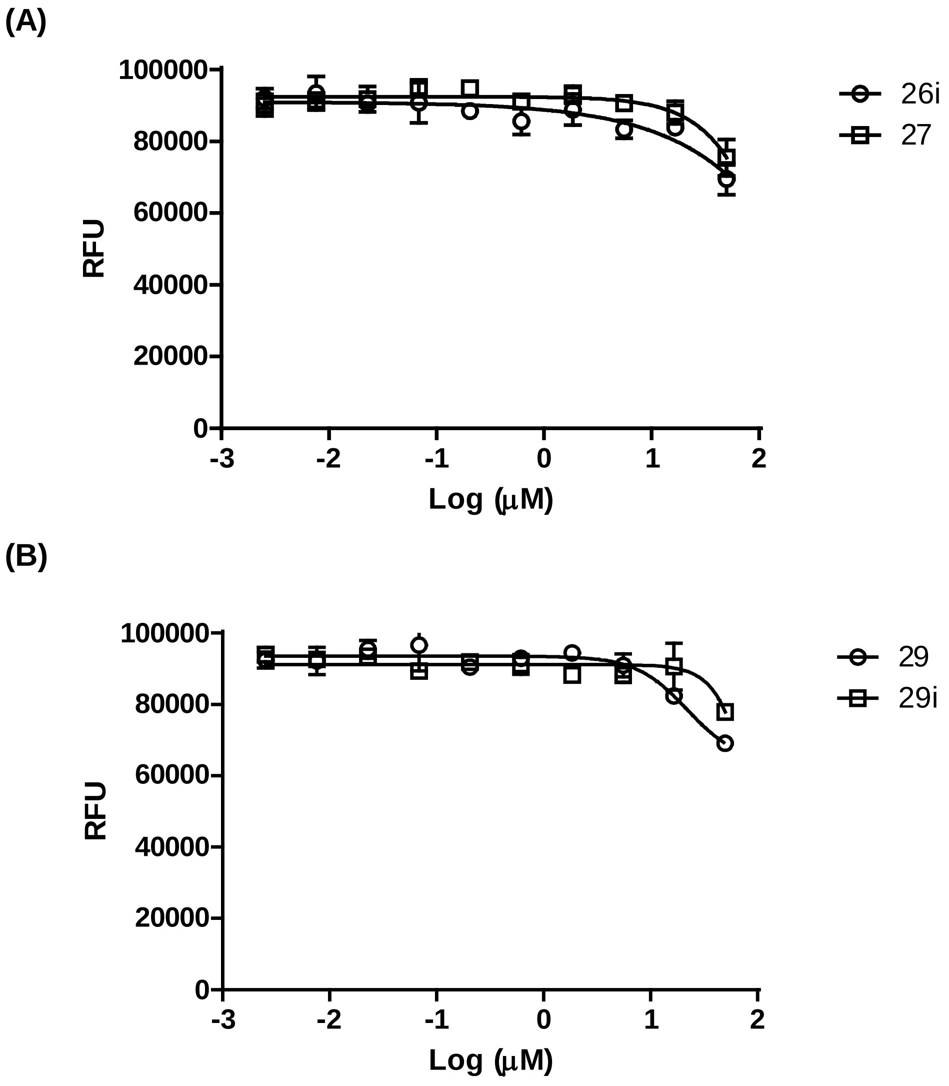

Table 1. Data collection and refinement statistics of DENV3 FL NS5 co-crystals.

PDB code	5JJR	5JJS
Crystallization conditions	18 % PEG 3350, 0.2 M Mg Acetate, 1 mM TCEP	18 % PEG 3350, 0.2 M Mg Acetate, 1 mM TCEP
Wavelength (Å)	1.0	1.0
Resolution (Å)	50-1.99 (2.11-1.99)	50-1.65 (1.75-1.65)
Space group	$P2_12_12$	$P2_12_12$
Unit cell (Å)	94.6/150.4/69.1	94.7/150.2/68.5
Measured reflections	44525 (64401)	784305 (127110)
Unique reflections	67993 (10422)	225431 (36133)
Multiplicity	6.5 (6.1)	3.5 (3.5)
Completeness (%)	99.0 (95.2)	99.2 (98.3)
Mean I/signa I (*I*)	10.04 (3.01)	13.13 (1.65)
Rmerge (%)	19.0 (58.6)	6.01 (74.0)
CC (1/2) (%)	99.2 (83.5)	99.9 (64.3)
R-work (%)	17.9	17.1
R-free (%)	22.5	19.8
Number of non-hydrogen atoms		
Macromolecule	6858	6920
Ligands	34 (L3) / 26 (SAH)	33 (L9) / 26 (SAH)
Solvent	473	628
Protein residues	845	
RMS (bonds) (Å)	0.010	0.010

RMS (angles) (°)	1.09	1.01
Ramachandran favoured (%)	99.3	99.3
Ramachandran outliers (%)	0.7	0.7
Average B-factor (Å^2)		
Macromolecules	23.2	26.9
Ligands	23.8	33.5
Solvent	28.2	35.9

Values in parenthesis are those for the last (highest resolution) shell

MR: Molecular replacement

CC1/2= percentage of correlation between intensities from random half-dataset [7]

Table 2. Analysis of DENV2 (strain NGC) WT and mutant replicons and virus replication.

(A) Absolute relative renilla luciferase light units (RLU) measured using renilla luciferase kit (Promega, USA) from BHK-21 cells at days 1-4 after electroporation with DENV2 WT and mutant replicons IVT RNA. Levels of secreted DENV2 (pfu/ml) were determined by plaque assay from supernatants of BHK-21 cells at days 1-4 after electroporation with DENV2 WT and mutant infectious viral IVT RNA.

	Renilla luciferase levels in DENV2 replicon (RLU)				DENV2 levels (pfu/ml) in culture supernatant			
Post-electroporation (hr)	WT	L511V	E802D	L511V/ E802D	WT	L512V	E802D	L512V/ E802D
24	2276562.9 ± 14766.5	3061250.9 ± 42578.2	684487.8 ± 9525.1	2323574.8 ± 66485	52500	1200	14000	65000
48	1701190.5 ±105595.8	2028336.0 ± 136472	1084181 ± 44392.6	1718929.8 ± 25087.1	132500	55000	425000	775000
72	458693.7 ± 278.6	618389.6 ± 2494	166496.1 ± 577.4	467361.7 ± 12258.4	825000	200000	4500000	5250000
96	60752.2 ± 1488.5	141721.1 ± 5521.3	19744.6 ± 1073	67222.3 ± 9150	7000000	3250000	9750000	3750000

(B) Mutant DENV2 replicons or virus replication measured by renilla luciferase levels or secreted virus titers, respectively, compared to WT replicon or virus levels (expressed as fold increases over WT levels).

	Renilla luciferase levels, fold increase compared to WT replicon levels			Fold increase in DENV2 levels (pfu/ml) in culture supernatant compared to WT DENV2 levels		
Post-electroporation (hr)	L511V	E802D	L511V/ E802D	L511V	E802D	L511V/ E802D
24	26.72	6.0	19.87	0.02	0.27	1.24
48	3.38	1.80	2.83	0.42	3.21	5.85
72	7.14	1.92	5.30	0.24	5.45	6.36
96	14.47	2.02	6.20	0.46	1.39	0.54

References

1. Niyomrattanakit P, Wan KF, Chung KY, Abas SN, Seh CC, Dong H, et al. (2015) Stabilization of dengue virus polymerase in de novo initiation assay provides advantages for compound screening. Antiviral Res 119: 36-46.
2. Ng C, Gu F, Phong WY, Chen Y, Lim SP, Davidson A, et al. (2007) Construction and Characterization of a Stable Subgenomic Dengue virus type 2 Replicon System for antiviral compound and siRNA screening. *Antiviral Res.* 76(3):222-231.
3. Blight KJ, Kolykhalov AA, Rice CM. 2000. Efficient initiation of HCV RNA replication in cell culture. *Science* 290(5498):1972–1974.
4. Yin Z, Chen YL, Schul W, Wang QY, Gu F, Duraiswamy J, et al. (2009) An adenosine nucleoside inhibitor of dengue virus. *Proc Natl Acad Sci USA* 106(48):20435-20439.
5. Wang QY, Dong H, Zou B, Karuna R, Wan KF, Zou J, et al. (2015) Discovery of Dengue Virus NS4B Inhibitors. *J Virol.* 89(16):8233-8244.
6. Zhao Y, Soh S, Zheng J, Chan KWK, Phoo WW, Lee CC, et al. (2015). A crystal structure of the dengue virus NS5 protein reveals a novel inter-domain interface essential for protein flexibility and virus replication. PLOS Pathogens 11(3):e1004682.
7. Karplus PA, Diederichs K. (2012). Linking crystallographic model and data quality. Science 336, 1030–1033.
8. Sievers F, Wilm A, Dineen DG, Gibson TJ, Karplus K, Li W, et al. (2011). Fast, scalable generation of high-quality protein multiple sequence alignments using Clustal Omega. Molecular Systems Biology 7: 539.
9. Molina MD, Jafari R, Ignatushchenko M, Seki T, Larsson EA, Dan C, et al. (2013) Monitoring drug target engagement in cells and tissues using the cellular thermal shift assay. Science 341: 84-7.
